# Spinal cord stimulation in chronic pain: evidence and theory for mechanisms of action

**DOI:** 10.1186/s42234-019-0023-1

**Published:** 2019-06-28

**Authors:** Jacob Caylor, Rajiv Reddy, Sopyda Yin, Christina Cui, Mingxiong Huang, Charles Huang, Ramesh Rao, Dewleen G. Baker, Alan Simmons, Dmitri Souza, Samer Narouze, Ricardo Vallejo, Imanuel Lerman

**Affiliations:** 1 Department of Anesthesiology, Center for Pain Medicine, University of California San Diego School of Medicine, La Jolla, CA USA; 2 VA Center of Excellence for Stress and Mental Health, VA San Diego Healthcare System, La Jolla, CA USA; 3 Department of Radiology, University of California San Diego School of Medicine, La Jolla, CA USA; 4 Department of Radiology, VA San Diego Healthcare System, La Jolla, CA USA; 5 Department of Bioengineering, Stanford University, Palo Alto, CA USA; 6 Department of Electrical and Computer Engineering, University of California San Diego, La Jolla, CA USA; 7 Department of Psychiatry, University of California San Diego School of Medicine, La Jolla, CA USA; 8 Center for Pain Medicine, Western Reserve Hospital. Department of Surgery, Northeast Ohio Medical School (NEOMED), Athens, OH USA; 9 Basic Science Research, Millennium Pain Center, Bloomington, IL USA; 10 School of Biological Sciences, Illinois State University, Normal, IL USA; 11 Department of Psychology, Illinois Wesleyan University, Bloomington, IL USA; 12 Present Address: VA San Diego, 3350 La Jolla Village Dr, (MC116A), San Diego, CA 92161 USA

**Keywords:** Spinal cord stimulation, Biomarker, Neurophysiology, Chronic pain, Complex regional pain syndrome, Failed back surgery syndrome, Mechanisms of action, Neuropathic pain, Objective measures, Neuroinflammation

## Abstract

Well-established in the field of bioelectronic medicine, Spinal Cord Stimulation (SCS) offers an implantable, non-pharmacologic treatment for patients with intractable chronic pain conditions. Chronic pain is a widely heterogenous syndrome with regard to both pathophysiology and the resultant phenotype. Despite advances in our understanding of SCS-mediated antinociception, there still exists limited evidence clarifying the pathways recruited when patterned electric pulses are applied to the epidural space. The rapid clinical implementation of novel SCS methods including burst, high frequency and dorsal root ganglion SCS has provided the clinician with multiple options to treat refractory chronic pain. While compelling evidence for safety and efficacy exists in support of these novel paradigms, our understanding of their mechanisms of action (MOA) dramatically lags behind clinical data. In this review, we reconstruct the available basic science and clinical literature that offers support for mechanisms of both paresthesia spinal cord stimulation (P-SCS) and paresthesia-free spinal cord stimulation (PF-SCS). While P-SCS has been heavily examined since its inception, PF-SCS paradigms have recently been clinically approved with the support of limited preclinical research. Thus, wide knowledge gaps exist between their clinical efficacy and MOA. To close this gap, many rich investigative avenues for both P-SCS and PF-SCS are underway, which will further open the door for paradigm optimization, adjunctive therapies and new indications for SCS. As our understanding of these mechanisms evolves, clinicians will be empowered with the possibility of improving patient care using SCS to selectively target specific pathophysiological processes in chronic pain.

## Background

Chronic Pain is a heterogenous, complex syndrome with significant burden for both the patient and the healthcare system. While the advent of multi-modal and multidisciplinary treatment approaches has improved strategies for chronic pain management, the push for opiate-free therapies has inspired development of novel approaches to both nociceptive and neuropathic pain syndromes. The gate-control theory of pain proposed by Melzack and Wall in 1965 spurred development of conventional Spinal Cord Stimulation (SCS), first surgically implanted in 1967 by Shealy, who noted that paresthesia elicited by electrical stimulation of the dorsal columns (DC) inhibited deep pain due to metastatic lung cancer (Melzack & Wall, 1965; Shealy et al., 1967). This groundbreaking work into the field of bioelectronic medicine ultimately opened the door for SCS and the rise of targeted neuromodulation.

SCS has been successfully utilized over the last half century in the adjunctive treatment of refractory pain syndromes not amenable to conservative therapy. Though the gate-control theory initially postulated a reduction in nociceptive pain in response to SCS, clinical experience demonstrated that patients with neuropathic and other rarer pain syndromes also received benefit. Current indications for SCS include Failed Back Surgery Syndrome (FBSS), Chronic Regional Pain Syndrome (CRPS), neuropathic pain, visceral abdominal pain and intractable angina pectoris (Lindblom & Meyerson, 1975; Linderoth & Foreman, 2017). While paresthesia-based SCS (P-SCS) now represents the traditional approach to neuromodulation of these dysregulated pain pathways, novel SCS paradigms and new anatomical targets have rapidly entered clinical use in the field of bioelectronic medicine. These include Dorsal Root Ganglion stimulation (DRG-S) as well as the paresthesia-free SCS (PF-SCS) paradigms: Burst SCS (B-SCS) and High Frequency SCS (HF-SCS). An additional PF-SCS paradigm, Evoked Compound Action Potential SCS (ECAP-SCS) has recently been developed, utilizing closed-loop monitoring to improve charge delivery to spinal targets. Together, the rapid clinical implementation of these novel paradigms has outpaced the bandwidth of the preclinical sciences to decipher the mechanisms and recruited pathways responsible for their efficacy. While some work has been completed, the trajectory of this ascension has left a sizeable knowledge gap between the basic sciences and clinical research, necessitating a review of evidence and theory for the mechanisms of action of these devices. In this review, we will 1) elucidate pertinent dysregulated pathways responsible for chronic pain syndromes, 2) identify the neuroanatomical targets thought to be modulated by SCS paradigms, 3) discuss proposed mechanisms of P-SCS, PF-SCS, DRG-S, and ECAP-SCS, and 4) present and evaluate the preclinical and clinical evidence in the context of these proposed mechanisms. Collectively, this review serves to recognize gaps in current knowledge and identify potentially rich avenues for future investigation.

## Parameters for spinal cord stimulation

**Fig. 1:**
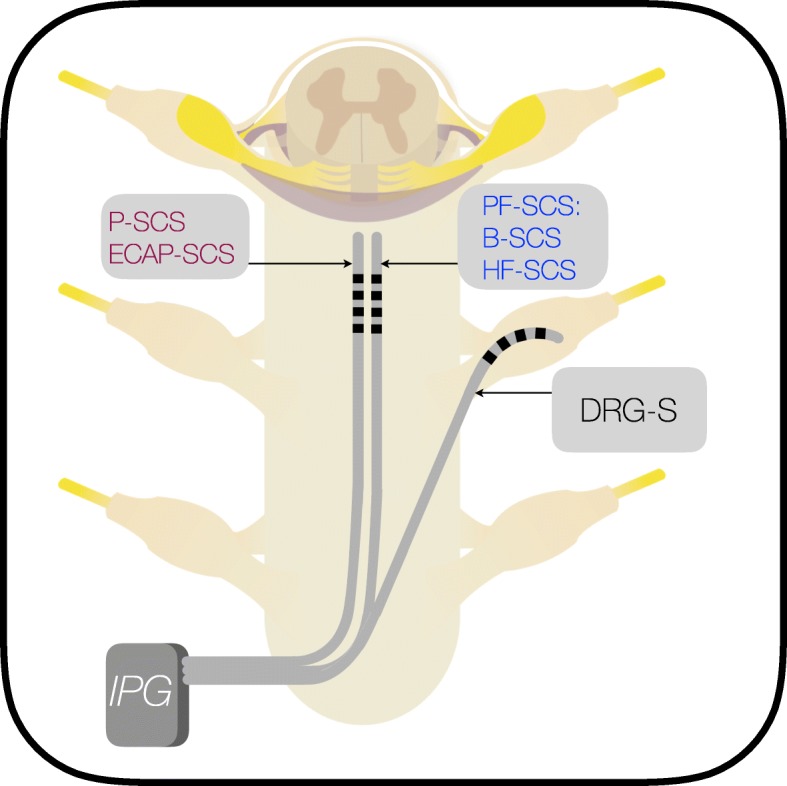
Lead Placement in P-SCS and PF-SCS: Dorsal column stimulation with traditional P-SCS, B-SCS and HF-SCS are anatomically placed over the dorsal columns. DRG-S is placed within the targeted foramina overlying the dorsal root ganglion. In all cases SCS can result in orthodromic activation or antidromic activation. Acronyms: IPG (Implantable Pulse Generator)

**Fig. 2:**
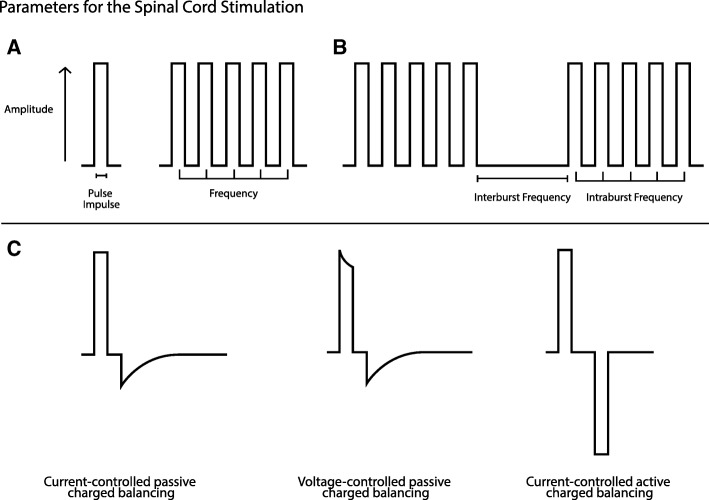
Parameters for Spinal Cord stimulation: Panel **a**: Amplitude: the peak current delivered, measured in milliamperes (mA). This impacts the number of fibers recruited and intensity of paresthesia. Amplitudes that are subthreshold do not generate an action potential and thus do not create paresthesia. Pulse Width (PW): the time over which the current is delivered, measured in microseconds (μs). The PW determines the amount of charge delivered for a given amplitude. Mathematical integration of the charge waveform yields the total charge delivered per pulse, measured in nanocoulombs. Increases in PW may recruit additional Aβ Fibers and broaden the area of paresthesia. Frequency: the number of pulses per second, measured in hertz (Hz). Panel **b**: Burst SCS parameters describing inter-burst frequency, or the number of bursts per second, and intraburst frequency, describing the number of pulses within a burst, measured in Hz. Panel **c**: The waveform or shape of the pulse can be divided into two segments: depolarization, or deflection above electroneutrality, and repolarization, the return to baseline. The depolarization waveform is determined by whether the system delivers the pulse in a Current-Controlled (CC) or Voltage-Controlled (VC) fashion. Current describes the flow of charge whereas voltage describes the potential difference between electrodes

Prescribed by the device generator, the implanted SCS leads deliver a charge “dose” to the target tissue. This generates a local electric field (EF). Neural bodies, synapses and axons which project through the EF may be modulated (Miller et al., 2016). While leads from P-SCS and PF-SCS enter the epidural space and deliver charge to the midline structures, DRG-S leads enter the epidural space, exit the neuroforamina and deliver charge to the adjacent DRG (Fig. 1) (Miller et al., 2016). The fundamental unit of a SCS program is the pulse, which is defined by the amplitude, pulse width (PW) and delivery waveform (Fig. 2, Panel a). By convention, frequency describes the number of pulses within one second (Miller et al., 2016). B-SCS utilizes a novel firing pattern, wherein stacked pulses are delivered followed by a period of quiescence which is then repeated. Thus, B-SCS can also be defined by intra-burst frequency and inter-burst frequency (Fig. 2, Panel b) (Chakravarthy et al., 2018a). Parameters for SCS charge delivery are reviewed in Fig. 2.

Lead impedance represents the inherent resistance of tissue to changes in charge. In SCS, the impedance is determined by the interaction between the lead contact and target, which may vary in an acute or chronic manner. Acute alterations in impedance reflect alterations in the anatomic relationship of the lead to the DC. These include lead migration, spine position such as lumbo-thoracic extension or abrupt changes in cerebrospinal fluid (CSF). Also caused by CSF volume expansion, local tissue fibrosis and scarring are thought to contribute more significantly to chronic elevations in impedance (Abejon & Feler, 2007). Interestingly, Kramer et al. (2015) noted these impedance changes are mitigated with close lead contact and use of the relatively immobile DRG-S (Kramer et al., 2015). The charge delivery strategy determines whether voltage or current will be fixed in relation to the system’s impedance (Fig. 2, Panel c). In current-controlled (CC) systems, the current is set; variations in impedance cause a change in voltage. In voltage-controlled (VC) systems, the voltage is set; variations in impedance cause a change in current (Miller et al., 2016; Schade et al., 2010). These systems will deliver a pulse with either constant voltage or constant current for a specified period of time (PW). The repolarization waveform is determined by the charge-balancing (CB) strategy (Fig. 2, Panel c) (Miller et al., 2016). Active CB creates a symmetric, biphasic pulse. Passive CB yields an asymmetric pulse. In either CB strategy, the initial pulse is followed by equal and opposite current movement to return the net charge to baseline; this avoids buildup of charge in the tissue, which may lead to injury if allowed to accumulate (Miller et al., 2016).

In response to a single pulse, axons and cell bodies within the EF exhibit a spectrum of modulation including no change, sub-threshold depolarization or generation of a mature action potential (AP). Intermittent, repeated pulses can induce summative APs. The susceptibility of fibers to depolarization is dependent on fiber thickness, myelination and distance from the lead. This susceptibility is summarized by each fiber’s strength-duration curve. As the total charge delivery of each pulse is the product of amplitude and PW, this curve represents total charge delivery necessary for AP generation (Abejón et al., 2015; West & Wolstencroft, 1983). Central to the perception of paresthesia in P-SCS, threshold depolarization of a DC Aβ fiber generates an AP, which may travel in an orthodromic and antidromic manner (West & Wolstencroft, 1983; Holsheimer, 2002). The coverage and intensity of paresthesia produced by P-SCS are determined by the amplitude and PW. Higher amplitude currents or voltages provide stronger paresthesia. Increasing the PW leads to greater fiber recruitment and increases the distribution of paresthesia (Holsheimer, 2002; Lee et al., 2011; Hershey et al., 2010). Frequency also plays a role in determining PW, as they are inversely related: as frequency increases, the available PW decreases. Additionally, as neurons intrinsically have maximal firing frequencies due to refractory periods, providing an overdriving stimulus would not allow every threshold pulse to generate an AP. Neuromodulation can occur without generating a paresthesia when the generated pulse is below the AP threshold of the fiber, as in PF-SCS paradigms that utilize properties of the strength-duration curve. The shape of this inverse hyperbolic curve can be exploited such that very large quantities of charge can be delivered without generating a paresthesia-evoking AP, especially at the extremes of PW and amplitude (Miller et al., 2016; Hershey et al., 2010). Subthreshold depolarizations by a weak EF are postulated to modulate neural networks by causing firing desynchrony, AP inhibition and changes in the resting membrane potential (Miller et al., 2016). Novel PF-SCS such as HF-SCS utilizes a charge delivery strategy such that a single, limited DC AP is generated only when PF-SCS is initiated, though no paresthesia is reported by the patient. These paradigms likely provide analgesia through novel mechanisms which remain to be elucidated and will be reviewed in their respective sections (Crosby et al., 2016). Despite advances in P-SCS and PF-SCS program settings, the common basis for classification of SCS remains frequency, with the exception of DRG-S which is an anatomic specification, and ECAP-SCS, which is a novel program (Miller et al., 2016). While this makes device categorization simple in colloquial contexts, in reality all pulse parameters must be considered to influence the physiologic response to SCS (Miller et al., 2016). Below, we will discuss the dysregulated pain pathways thought to be modulated by the charge delivery of SCS.

## Mechanisms of hyperalgesia and allodynia

Pain is a context-dependent sensory or emotional experience associated with actual or potential damage to tissue. A peripheral nociceptive stimulus is perceived via pathways projecting to cortical and subcortical regions, resulting in the conscious and affective pain experience. Chronic pain is a product of the dysregulation in pain processing in response to peripheral or central injury. Serving to guide clinical therapies, preclinical models attempt to recreate these dysregulated pain pathways, further our understanding of antinociceptive mechanisms and have spurred the development of novel SCS paradigms. Animal models of hyperalgesia and allodynia include chronic constriction injury (CCI), spinal nerve ligation (SNL) and spared nerve injury (SNI); these models generate an incomplete nerve injury representative of typical human pathology, in opposition to complete transection models (Todd, 2010). SNI and SNL animals develop tactile allodynia within the first day, whereas CCI animals develop allodynia over the course of one week. The target of the CCI and SNI models are the sciatic nerves whereas SNL targets the L4/L5 spinal nerves (Todd, 2010). Measuring paw withdrawal thresholds (PWT) to evaluate pain behavior before and after intervention, animal models are routinely employed in the assessment of SCS paradigms and elucidation of their respective mechanisms. At the crux of chronic pain pathogenesis is central sensitization (CS), which the IASP Task Force defines as “increased responsiveness of nociceptive neurons in the central nervous system (CNS) to normal or subthreshold afferent input” (Sandkuhler, 2010; Taxonomy ITFo, 1994). Peripheral injury leads to pathologic activation of post-synaptic nociceptive projection neurons (PN) and has become the focus of research in animal models (Sandkuhler, 2009). SCS-mediated pain relief is thought to act through modulation of the maladaptive aggregate response to local injury, neuroinflammation and CS at both the segmental and supraspinal levels, processes which are hallmarks of hyperalgesia and allodynia. The ligand-receptor pairs of interest to the process of CS include: 1) Glutamate, which activates N-methyl-D-aspartate (NMDA), amino-3-hydroxy-5-methyl-4-isoxazole propionate (AMPA), and metabotropic glutamate (mGLU) receptors (Todd, 2010; Latremoliere & Woolf, 2009), 2) Substance P (SP), which activates Neurokinin-1(NK1) Receptors (Latremoliere & Woolf, 2009), 3) Brain-derived neurotrophic factor (BDNF) which activates Tropomyosin receptor kinase B (TrkB) receptor (Latremoliere & Woolf, 2009), 4) Calcitonin Gene-related Peptide (CGRP) which activates CGRP Receptor, 5) Gamma-aminobutyric Acid (GABA) which activates GABA Receptors, including the GABA A (GABA_A_) Receptor and GABA B (GABA_B_) Receptor and 6) Glycine (Gly) which activates the Gly Receptor (GlyR).

**Fig. 3:**
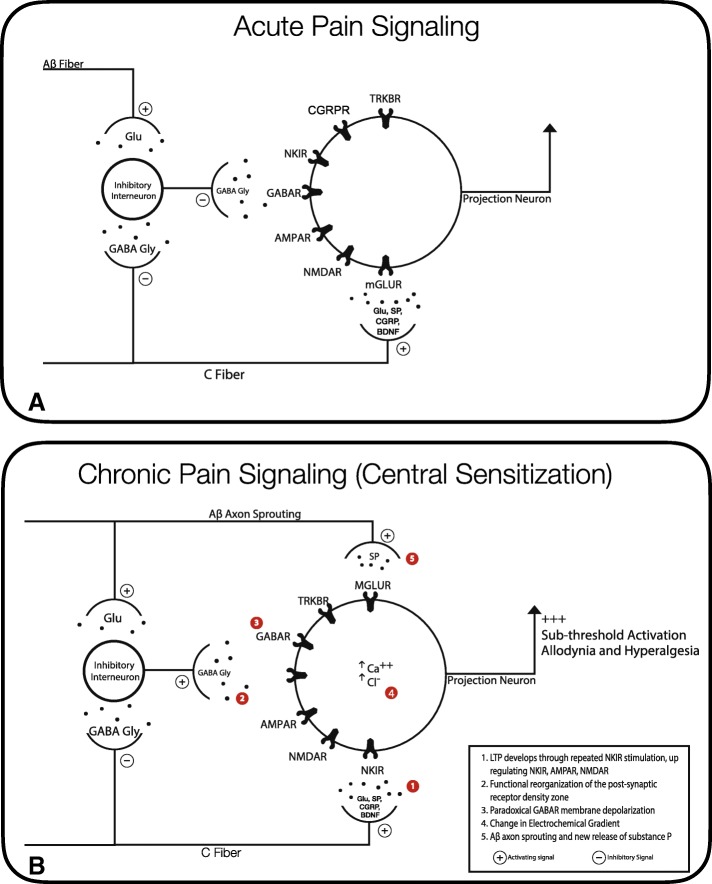
Mechanisms of Hyperalgesia and Allodynia. Panel **a**) Non-pathologic nociception whereby C-fiber and Aβ inputs relay through an interneuron to modulate ascending signals via the projection neuron. Panel **b**) Nerve injury or repeated peripheral c-fiber stimuli leads to central sensitization through multiple mechanisms including changes in receptor kinetics, resting membrane potential and phenotypic transformation of Aβ afferent fibers. Through long term potentiation and altered receptor expression at the post receptor density zone, subthreshold stimulation evokes action potentials leading to classic hyperalgesia. Aβ axon sprouting after injury and secretion of substance P may offer further explanation for the development of tactile allodynia. Acronyms: Glu (Glutamate), Gly (Glycine), GABA (gamma amino butyric acid), SP (Substance P), BDNF (Brain Derived Neurotrophic Factor), CGRP (Calcitonin Gene Related Peptide), MGLUR (metabotropic glutamate receptor), TRKB (Tropomyosin receptor kinase), GABAR (GABA Receptor), AMPAR (AMPA Receptor), CGRPR (CGRP Receptor), NMDAR (NMDA Receptor), NK1R (Neurokinin-1 Receptor), LTP (Long Term Potentiation)

CS is thought to result as a consequence of changes to the neuroactive milieu and post-synaptic receptor activation state. Pathologic nociception develops after CS through long-term potentiation (LTP), loss of spinal inhibition, neural plasticity and phenotypic transformation of low-threshold Aβ afferents (Todd, 2010). Windup represents the short-term, reversible temporal summation of slow C-fiber activation of NK-1 and CGRP receptors, leading further to NMDA receptor activation and prolongation of progressive membrane depolarization (Latremoliere & Woolf, 2009). Though windup is commonly ascribed to the pathogenic process of CS, LTP produces durable activation changes of the NK1-positive PN. Repetitive stimulation of NK1 positive neurons leads to synaptic amplification through a Ca^2+^-dependent mechanism (Latremoliere & Woolf, 2009; Ikeda et al., 2006). Implicated in CS, repetitive stimulation leads to elevations in intracellular calcium. Activation of Ca^2+^-dependent protein kinases leads to receptor ionophore phosphorylation and decreased potassium currents, resulting in increased receptor efficacy and potentiation of AMPA, NMDA and NK1 signaling (Sandkuhler, 2009; Latremoliere & Woolf, 2009). Similar to excitatory LTP, nerve injury can also result in dysregulation of inhibition, primarily through a functional loss of GABA signaling. Moreover, after nerve injury, PN may become altered so that GABA_A_ and glycine receptor signaling becomes excitatory. As chloride ionophores, GABA_A_ and glycine receptors facilitate Cl^−^ movement across its transmembrane gradient, which is maintained by active Cl^−^ transporters. Noted after nerve injury, a reduction in Cl^−^ exporter function yields an increase in intracellular Cl^−^ concentration (Benzon et al., 2014). Transmembrane gradient reversal may lead to paradoxical AP generation with the activation of GABA_A_ and glycine receptors, whereby an initially inhibitory or non-noxious stimulus now generates a nociceptive AP (Benzon et al., 2014). This mechanism, in part, also explains the paradoxical nociceptive response to Aβ drive, while pathological ectopic Aβ sprouting also contributes (Benzon et al., 2014). While the pathogenesis of CS is a heterogenous process, changes in receptor kinetics leading to sub-threshold and paradoxical AP generation are one explanation for hyperalgesia and allodynia (Fig. 3). Paramount, modulation of CS at the segmental level remains the mechanistic cornerstone of analgesia from SCS. In the non-pain state, inhibitory interneurons release GABA and glycine to suppress postsynaptic depolarization through Cl^−^ dependent hyperpolarization (Benzon et al., 2014). In nerve-injury models, loss of GABAergic positivity in the dorsal horn (DH) has been observed with CS, due to cell death or the depletion of GABA in terminals (Moore et al., 2002; Polgar et al., 2003). GABA depletion is known to play a significant role in CS, as allodynia is reduced with intrathecal GABA_A_ receptor agonism and induced by nonspecific GABA receptor antagonism (Hwang & Yaksh, 1997; Malan et al., 2002). Whereas GABA depletion, receptor potentiation and ionophore kinetics offer competing hypothesized mechanisms of hyperalgesia and allodynia, the CS phenotype is likely oversimplified in clinical practice (Fig. 3).

Reorganization and phenotypic transformation of mechanoreceptive Aβ afferents also plays a role in the pathogenesis of CS. Woolf et al. (1992) demonstrated that the central terminals of Aβ afferents, normally projecting to laminae III and IV, sprouted to lamina II after axotomy (Woolf et al., 1992). This arborization of non-nociceptive, low-threshold myelinated inputs to lamina II primarily populated by nociceptive terminals likely also contributes to tactile allodynia after nerve injury (Woolf et al., 1992). Moreover, Aβ afferents began expressing SP after nerve injury, thought to contribute to allodynia (Fig. 3, Panel b) (Hughes et al., 2007; Neumann et al., 1996). Lastly, abnormal polysynaptic circuits may lead to the miscoding of a mechanoreceptive input as nociceptive (Schoffnegger et al., 2008). While initially it was thought that SCS activation of Aβ afferents would inhibit ascending nociceptive inputs through the simplified gate-control mechanism, it is now clear that additional mechanisms at the segmental and supraspinal levels play a role in SCS-mediated analgesia. Offering an explanation for the variable response to SCS, seemingly similar clinical phenotypes may be generated by vastly different pathologic mechanisms, explained by the heterogenous, maladaptive process of central sensitization.

## Neuroanatomical targets of spinal cord neuromodulation

### Spinothalamic tract

A functional understanding of the spinothalamic tract (STT) is critical to evaluating mechanisms of SCS-mediated analgesia. AP originating from the stimulation of peripheral nociceptors are transmitted to the CNS via the thinly myelinated Aδ and unmyelinated C fibers (Benzon et al., 2011). Aδ fibers, conducting at a moderate velocity of 5–10 m/s, synapse in Rexed laminae I and III-V of the spinal cord DH (Benzon et al., 2011). As Aδ fibers conduct more quickly than C fibers, they are considered the first pain signal, carrying acute pain, temperature and pressure (McMahon et al., 2013). C fibers, conducting at a slower velocity of < 2 m/s, synapse superficially in Rexed laminae I-II. AP to C fibers are initiated by multiple receptor types, including thermoreceptors, mechanoreceptors and chemoreceptors; thus these fibers are classified as polymodal (Todd, 2010). C fibers carry the delayed pain response, often characterized as poorly-localized burning or aching. By synapse quantity, Aδ and C fibers primarily project to interneuron circuitry, which serves as a conduit to conduct inhibitory or excitatory signals to ascending PN (Polgar et al., 2008). However, primary afferents also synapse directly on two types of second-order PN: multimodal Wide Dynamic Range (WDR) neurons and Nociceptive Specific (NS) neurons (Todd, 2010; McMahon et al., 2013). WDR neurons, concentrated in laminae III-V, receive input from interneurons of Aβ, Aδ and C fiber origin (Todd, 2010; McMahon et al., 2013). Because they receive inputs from both noxious and non-noxious stimuli, it is fitting that WDR neurons have a graded increase in firing frequency and amplitude with repetitive polymodal stimuli (Mendell, 1966). NS neurons, concentrated superficially in laminae I-II, only receive input from fibers carrying noxious stimuli and do not exhibit a graded response to pain stimuli. Moreover, stimulation of independent sympathetic neurons shows significant activation of WDR neurons while NS neurons are not as impacted (Roberts & Foglesong, 1988). Axons of WDR and NS neurons carrying crude touch, pain and temperature decussate and ascend in the anterolateral STT, synapsing on nuclei in the posterior, medial and lateral thalamus. Collateral axons ascend and project onto centers for autonomic regulation and somatosensory modulation within the brainstem and midbrain. A functional understanding of this tract is critical to evaluating SCS mechanisms as it serves as the primary modulatory target via both segmental spinal and supraspinal mechanisms.

### Dorsal column – medial lemniscus pathway

**Fig. 4:**
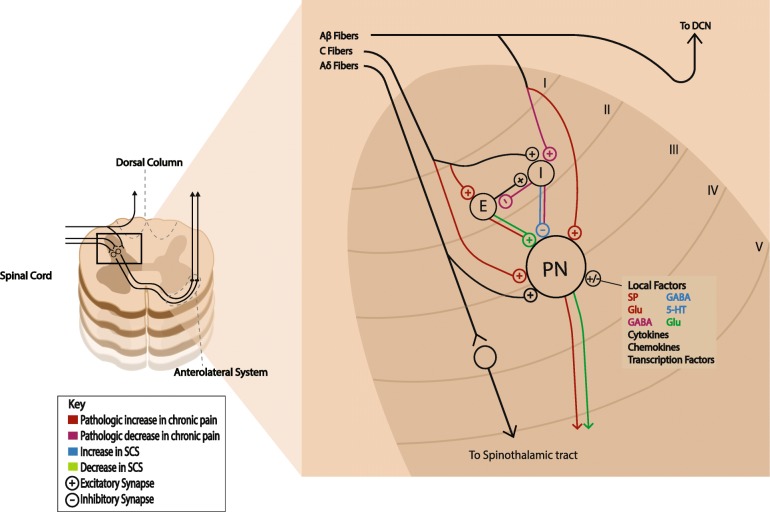
Changes in Dorsal Horn Circuitry to chronic pain and SCS. In the development of chronic pain, neural circuitry undergoes rewiring wherein abnormal enhancement of excitatory pathways and loss of inhibition facilitate nociceptive transmission to sub-threshold stimuli. Increased excitatory interneuron input, decreased inhibitory interneuron input and local factors contribute to pathologic destabilization of normal input balance to the projection neuron. SCS changes the balance of nociceptive and antinociceptive inputs through the activation of local segmental and descending supraspinal mechanisms to in-part restore balance to this network. Acronyms: DCN (Dorsal Column Nuclei), E (Excitatory Interneuron), I (Inhibitory Interneuron), PN (Projection Neuron), SP (Substance P), Glu (Glutamate), GABA (Gamma Aminobutyric Acid), 5-HT (5-Hydroxytryptamine, Serotonin)

**Fig. 5:**
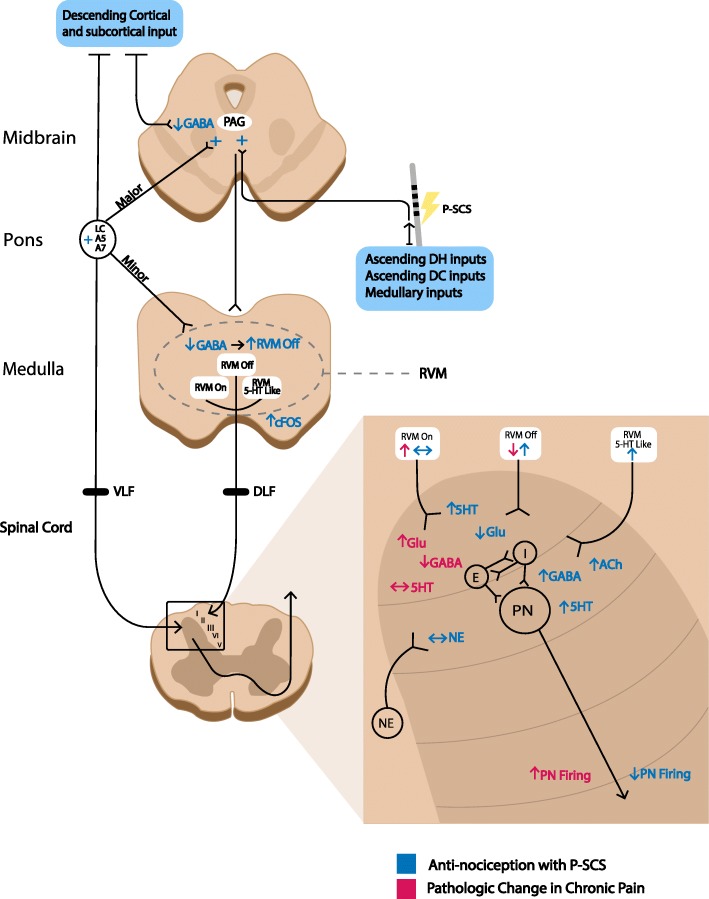
Supraspinal Mechanisms of Spinal Cord Stimulation. A hallmark of chronic pain, abnormal enhancement of excitatory pathways and a loss of inhibition facilitate nociceptive transmission to sub-threshold stimuli. With SCS, orthodromic activation of supraspinal centers of pain control facilitates antinociception through activation of the DAS, largely through recruitment of the PAG, RVM and LC. Increases in spinal ACh, 5-HT and GABA as well as decreased spinal glutamate with SCS are in part thought to be a result of descending pathway recruitment. ‘Up’ arrows represent increased concentration or activity, whereas ‘down’ arrows represent opposite. ‘Sideways’ arrows represent no change. Acronyms: PAG (Periaqueductal Gray), RVM (Rostral Ventromedial Medulla), LC (Locus Coeruleus), A5 (Noradrenergic Cell Group A5), A7 (Noradrenergic Cell Group A7), DH (Dorsal Horn), DC (Dorsal Column), VLF (Ventrolateral Funiculus), DLF (Dorsolateral Funiculus), RVM ON (RVM On cells projecting from the RVM to the DH), RVM OFF (RVM OFF cells projecting from the RVM to the DH), RVM 5-HT Like (RVM 5-HT Like cells projecting from RVM to DH), cFOS (proto-oncogene), E (Excitatory Interneuron), I (Inhibitory Interneuron), PN (Projection Neuron), Glu (Glutamate), 5-HT (5-hydroxytryptamine), ACh (Acetylcholine), NE (Norepinephrine), GABA (Gamma Aminobutyric Acid)

Though responsible for the paresthesia induced by direct stimulation in P-SCS, the DC normally carry fine touch, vibration and proprioceptive afferent inputs from peripheral mechanoreceptors (Todd, 2010). These peripheral APs enter the spinal cord via axons of pseudounipolar neurons at the DRG and ascend in the DC via the dorsal column medial lemniscus (DCML) pathway. As discussed previously, Aβ fibers also send inputs to circuits of the DH, affecting afferent nociceptive AP and contribute to CS. First order fibers ascending in the DCML then synapse on their respective nuclei within the ipsilateral medulla: the upper extremities synapse in nucleus cuneatus laterally, whereas the lower extremities synapse on the nucleus gracilis medially (McMahon et al., 2013). Second order axons decussate in the brainstem forming the internal arcuate fibers, then ascend cephalad through the brainstem and midbrain in the medial lemniscus, acquiring trigeminal inputs, ultimately synapsing on the Ventral Posterior Lateral (VPL) and Ventral Posterior Medial (VPM) nuclei of the thalamus. Third and higher order neurons carrying inputs from the DCML and STT project to the somatosensory cortex (McMahon et al., 2013). While the stimulation of DC Aβ fibers activates DH inhibitory circuits in an antidromic manner (Fig. 4), other important mechanisms contribute to SCS-mediated analgesia (Fig. 5).

### Native signal modulation: beyond gate control

The origins of Melzack and Wall’s gate-control theory of pain developed from the hypothesis that cells of the substantia gelatinosa (SG) played a role in presynaptic inhibition of cutaneous sensory input (Mendell, 2014). Based on their input mapping and the discovery of axo-axonal synapses, Szentagothai (1964) postulated that spinal interneurons played a role in modulating ascending nociceptive signals (Mendell, 2014; Szentagothai, 1964). Specifically, they hypothesized the existence of a *single interneuron* with inhibitory input to the small and large fiber terminals; this interneuron itself received stimulatory input from large fibers (Aβ) and inhibitory input from small fibers (Aδ and C) (Melzack & Wall, 1965; Mendell, 2014). Central to this theory, they postulated that an imbalance between small and large fiber input could lead to disinhibition of the WDR neuron, thus leading to transmission of the ascending pain signal. Support for this model was provided by experiments demonstrating presynaptic control in the SG by stimulating small or large fibers and measuring evoked dorsal root potentials (Mendell, 2014). Though the gate control theory of pain continues to serve as a simplified model for framing pain signaling, it is current cannon that many additional factors contribute to spinal nociceptive modulation. Relevant to SCS-mediated analgesia, local interneurons, descending projections, glia and neuroinflammation comprise the contributing SG architecture to nociceptive processing (Fig. 4).

### Spinal interneurons

Inhibitory and excitatory interneurons play an important role in the sensory signaling cascade, modulating the activity of WDR and NS neurons in the DH. Adding to Melzack and Wall’s gate-control theory, interneurons form an adaptive neural circuitry critical in modulation of afferent nociceptive signaling that establishes a balance between excitatory and inhibitory inputs. Laminae I-III of the DH are populated with a plexus of interneurons (Todd, 2010). Excitatory interneurons secreting glutamate comprise the non-GABAergic interneurons of laminae I-III, identified by immunostaining at the synaptic bouton (Todd, 2010; Todd et al., 2003). GABA and glycine immunostaining in the rat DH exposed a dense plexus of inhibitory axons that largely arise from local interneurons in laminae I-III (Polgar et al., 2003; Todd & Sullivan, 1990). As evidence for circuit plasticity, Keller et al. (2001) has demonstrated that inhibitory synapses undergo maturation and tuning through refinement of neurotransmitter release (Keller et al., 2001). Potentially, SCS-mediated analgesia contributes to restoration of non-pathologic equilibrium in this inherently plastic circuitry (Saadé et al., 2006; Lind et al., 2016). Attenuation of WDR neuron hyperexcitability through Aβ-mediated inhibitory control is one proposed mechanism of P-SCS analgesia (Simone et al., 1991; Willis et al., 1974; Chung et al., 1979). Careful neuroanatomical studies show that both SG interneurons and WDR PN are involved in GABAergic synapses (Todd, 2010; Zeilhofer et al., 2012; Lekan & Carlton, 1995). Single unit recording studies show dorsal column stimulation inhibits WDR neuron hyperexcitation within the DH deep laminae (Hillman & Wall, 1969; Lindblom & Meyerson, 1975; Foreman et al., 1976; Linderoth et al., 2009; Zhang et al., 2014). Critically, P-SCS applied to the DC prevented WDR sensitization secondary to C Fiber-induced LTP and wind-up (Wallin et al., 2003; Guan et al., 2010). Moreover, it has been observed that P-SCS inhibits WDR neuron activation in animal models of neuropathic pain (Yakhnitsa et al., 1999). Numerous preclinical studies have confirmed that Aβ fiber suppression of neuropathic pain is mediated through a GABAergic mechanism (Sivilotti & Woolf, 1994; Duggan & Foong, 1985). While studies demonstrating the effects of SCS on GABAergic inhibitory interneurons are reviewed in depth in the 'gamma-aminobutyric acid' section, it should be noted that suppression of PN firing by P-SCS is dependent on intact cord architecture and lead proximity (Hillman & Wall, 1969; Foreman et al., 1976; Smits et al., 2012). Taken together, there is strong evidence for segmentally-mediated DH regulation of nociception, either through potentiation or inhibition of PN by local interneurons. The relative contribution of segmental and supraspinal antinociceptive mechanisms mediated by either P-SCS or PF-SCS remains an intense area of research.

### Descending Antinociceptive systems

Paresthesia based spinal cord stimulation provided the first clear evidence that SCS activates the descending antinociceptive system (DAS), thus modulating the DH and PN. First, Nashold et al. (1972) suggested that SCS masked pain at the supraspinal level based on data showing that DC stimulation produced measurable EEG potentials consistent with known somatosensory evoked potentials (Nashold et al., 1972). While investigating local mechanisms of SCS, Foreman et al. (1976) further suggested presence of a supraspinal mechanism given that mid-thoracic and cervical stimulation depressed spinothalamic tract activity measured at the lumbosacral enlargement (Foreman et al., 1976). Clarifying supraspinal control over DH PN firing, Saade et al. (1985) utilized DC-transected decorticate-decerebrate cats, showing that DC stimulation rostral to the transection as well as direct stimulation of nucleus raphe magnus inhibited firing in DH neurons (Saade et al., 1985). Expanding on this observation, their group used carefully-designed brainstem lesioning experiments to elucidate the connections between the DC, periaqueductal gray (PAG) and nuclei of the reticular formations (Fig. 5) (Saade et al., 1982; Saade et al., 1983). In subsequent investigations in which they stimulated only the DC nuclei, their group confirmed a supraspinal inhibitory loop in an awake rat model (Saade et al., 1986). These studies accelerated the interest into DAS as exploitable mechanisms of SCS (Fig. 5).

Colloquially termed the serotonergic DAS, inputs to the PAG relay through the rostral ventromedial medulla (RVM), descend in the dorsal lateral funiculus (DLF) and project to interneurons of the DH (Fig. 5). While the serotonergic DAS largely utilizes the neurotransmitter serotonin (5-HT), opioidergic and GABAergic mechanisms contribute at the supraspinal and segmental levels (Cui et al., 1999). The PAG, a well-known opioidergic pain center, does not have direct projections to the DH, and instead relays its descending signals through the RVM (Behbehani & Fields, 1979). A spinal relay for all descending non-noradrenergic pain inhibition, the RVM is comprised of the nucleus raphe magnus and nuclei of the reticular formations (Newman, 1985). Both the PAG and RVM receive ascending inputs from DH PN, creating a feedback loop (Dong et al., 1999; Hardy, 1986; Helmstetter et al., 1998; Sakata et al., 1989). The PAG and RVM are highly innervated by pain modulating centers, receiving projections from cortical and subcortical structures, thus contributing the conscious, stress and emotional responses to pain (Dong et al., 1999; Hardy, 1986; Helmstetter et al., 1998; Sakata et al., 1989). Together, these supraspinal cortical and subcortical communications comprise the neural signature of pain, which will be evaluated in the context of P-SCS and PF-SCS in the 'SCS affects Cortical and subcortical pain processing' section.

Fibers from the RVM descend via the DLF to widely innervate the DH (Todd, 2010; Basbaum et al., 1976). RVM cells projecting to the DH have multiple classifications: ON, OFF, 5-HT like or neutral. While neutral cells and 5-HT like cells have only been partially characterized, more is understood about the firing patterns of ON and OFF cells (Fields, 2004). Normally quiescent, ON cells are activated by a nociceptive stimulus, enhancing DH nociceptive transmission (Mendell, 2014). Pathologic ON cell activity is thought to play a role in CS and opioid-induced hyperalgesia (Mendell, 2014). Tonically suppressing DH pain transmission, pathological OFF cell deactivation leads to increased pain transmission in the DH. Their activity enhanced by opioids, RVM OFF cell regulation of the nociceptor-PN synapse creates an opioid-dependent supraspinal pain gate (McMahon et al., 2013; Mendell, 2014). In the RVM, exogenously administered opioids activate OFF cells and suppress ON cells through a GABAergic mechanism (McMahon et al., 2013). Sharing a partial pathway with opioids, Song et al. (2013) has shown that P-SCS results in DH antinociception through activation of RVM OFF cells and 5-HT like cells, but has no effect on ON cells (Fig. 5) (Song et al., 2013a). Moreover, P-SCS mediated analgesia was attenuated with GABA receptor agonism but independent of opioidergic mechanisms within the RVM (Song et al., 2013a). Because opioids and P-SCS share a partially redundant mechanism, opioidergic mechanisms may not contribute to P-SCS mediated analgesia, discussed in the 'Endogenous Opioids' section.

The descending noradrenergic pathway modulates ascending pain signals through the release of norepinephrine (NE) in the DH, specifically laminae I-III of the SG. Axons from the locus coeruleus (LC) and associated cell bodies provide descending spinal NE, synapsing on nociceptive afferents and lamina II spinal interneurons (Kwiat & Basbaum, 1992). While the LC, A5 and A7 all receive inputs from the PAG, the LC receives additional notable cortical and sub-cortical relays (Bajic & Proudfit, 1999; Bernard et al., 1996; Cedarbaum & Aghajanian, 1978). Ascending signals originating in the DH send projections to the LC while redundant pathways relay from the insular cortices, amygdala and hypothalamus. This creates a neural circuit responsive to emotion and stress (Bernard et al., 1996; Gauriau & Bernard, 2002). It is likely that part of the analgesic effect from activation of the PAG is due to recruitment of the noradrenergic DAS (Cui et al., 1999; Bajic & Proudfit, 1999). Evidence for SCS modulation of the noradrenergic and serotonergic DAS is discussed below.

## Segmental and Supraspinal neurotransmitters

Preclinical pain models have served to clarify mechanisms contributing to SCS-mediated analgesia. From careful lesioning experiments to direct DH sampling using microdialysis catheters, changes in neurotransmitter content, concentration and synthetic enzyme function have molded our understanding of these pathways. However, differences in implementation, methodology and inability to isolate cell-specific contributions to neurotransmitter release makes it critical to constantly reassess our understanding of spinal and supraspinal pain mechanisms. Specifically, it is unclear the extent to which an observed neurotransmitter change can be ascribed to a particular mechanism, as the pathways remain incompletely elucidated. For example, the relative contributions of interneurons, glia, non-nociceptive fibers and DAS to GABA release in response to SCS remains unclear. While we recognize the limitations of experimental models, progress has been made with regard to clarifying these pathways. We therefore discuss current constructs of pain signaling in SCS as they relate to the specific neurotransmitter, site of action and proposed origin.

### Serotonin

Modulated by SCS and released in the DH, serotonin (5-hydroxytrypamine, 5-HT) is a monoamine neurotransmitter involved in the in the serotonergic DAS. A component of the RVM and a known serotonergic center, electrical stimulation of the nucleus raphe magnus led to GABA release in the DH, indicating that spinal 5-HT acts through a GABAergic intermediary (Kato et al., 2006; Tazawa et al., 2015). Interestingly, electrical stimulation of the PAG attenuated nociceptive inputs through a serotonergic mechanism, supporting that opioidergic mechanisms of analgesia also utilize this 5-HT DAS (Akil & Liebeskind, 1975; Liu et al., 1988). Preclinical pain models consistently demonstrate that descending 5-HT originates from the RVM and P-SCS models confirm this relationship (Vera-Portocarrero et al., 2006; Suzuki et al., 2004; Pertovaara, 2000; Newton & Hamill, 1988). While Peng et al. (1996) showed that analgesia from PAG stimulation was blocked by 5-HT_3_ receptor antagonists, Li et al. (2014) demonstrated that DH 5-HT resulting from P-SCS was exclusively synthesized in supraspinal nuclei and transported anterograde to nerve terminals (Peng et al., 1996; Li et al., 2014). Localizing the source of supraspinal 5-HT, Maeda et al. (2009) showed that P-SCS induced expression of c-Fos in the RVM (Maeda et al., 2009). Tazawa et al. (2015) further showed that the supraspinal nuclei and not the local spinal cord were responsible for production of 5-HT after P-SCS therapy. Moreover, they noted increased activation and number of serotonergic neurons in the dorsal raphe nucleus, a center adjacent to the ventral PAG with communications to the RVM and LC (Tazawa et al., 2015). Lastly, they noted that while both the serotonergic and noradrenergic DAS contributed to P-SCS-mediated analgesia, the 5-HT pathway played a greater role in antinociception (Tazawa et al., 2015). Together, these studies demonstrate activation of the serotonergic DAS in response to P-SCS therapy, which in turn innervates the DH, providing antinociceptive drive. Linderoth et al. (1992) first showed that DC stimulation increased 5-HT levels in the DH (Table 1) (Linderoth et al., 1992). Clarifying this observation, their group further demonstrated the presence of 5-HT staining nerve terminals in the SG and noted that intrathecal 5-HT potentiated the antinociceptive effects of P-SCS. Moreover, by showing that antinociception from P-SCS was attenuated with GABA_B_ receptor antagonism and unchanged with muscarinic 4 acetylcholine receptor (M4 mAChR) antagonism, they demonstrated the presence of a GABAergic link and accelerated interest into 5-HT receptor subtyping (Table 2) (Song et al., 2009). Through agonist-antagonist studies, Song et al. (2011) utilized a rat model of mononeuropathy to clarify the role of 5-HT and GABA receptor subtypes on analgesia from P-SCS therapy (Song et al., 2011). Specifically, they noted that agonists of 5-HT_2_ and 5-HT_3_ receptors enhanced P-SCS analgesia while the benefits from 5-HT_3_ receptor agonists were inhibited more with antagonism of GABA_B_ than GABA_A_ receptors. While they did not discover any difference with 5-HT_1_, 5-HT_6_ and 5-HT_7_ receptor antagonism, they noted that antagonism of 5-HT_2A_ and 5-HT_4_ receptors attenuated the P-SCS response to tactile hypersensitivity. In total, their work suggests that while 5-HT_2A_ and 5-HT_4_ receptor agonism contributes to analgesia, P-SCS works to a greater extent through 5-HT_3_ receptors with a GABA_B_ receptor link (Song et al., 2011). The preclinical evidence for neurotransmitter receptor subtype contribution to SCS-mediated analgesia is summarized in Table 2. These preclinical models indeed demonstrate that P-SCS activates the serotonergic DAS in an orthodromic manner (Fig. 5). However, the *clinical* magnitude of 5-HT contribution to SCS-mediated antinociception is still unknown. Nonetheless emerging reports support the construct that serotonergic mechanisms play a significant role in pain relief. Prabhala et al. (2019), demonstrated that Duloxetine, a 5-HT and NE reuptake inhibitor, combined with P-SCS therapy significantly improved pain scores at one year compared to SCS alone (Prabhala et al., 2019). Taken together, preclinical and emerging clinical investigations suggest the significance of P-SCS mediated orthodromic activation of the serotonergic DAS for analgesia. However, further work is needed to determine if 5-HT may play a role in other paradigms, such as PF-SCS or DRG-S (Tables 1 and 2).

**Table 1:** Measured Neurotransmitters in response to spinal cord stimulation (SCS)

	Tissue Sampled and Measured Change To:			
Neurotransmitter	P-SCS	B-SCS	HF-SCS	DRG-S
Glu	SC: ↓ ^(Cui et al., 1997)^ PAG: ↓ ^(Stiller et al., 1995)^			
5-HT	SC: ↑ ^(Linderoth et al., 1992; Song et al., 2009)^ PAG: ↔ ^(Stiller et al., 1995)^			
GABA	SC: ↑ ^(Cui et al., 1997; Stiller et al., 1996)^ PAG: ↓ ^(Stiller et al., 1995)^ RVM: ↓ ^(Song et al., 2013c)^ Plasma: ↑ ^(Crosby et al., 2015a)^	Plasma: ↔ ^(Crosby et al., 2015a)^		
NE	SC: ↔ ^(Song et al., 2013b)^ CSF: ↑ ^(Levin & Hubschmann, 1980; Liu et al., 2008)^ Plasma: ↑ ^(Levin & Hubschmann, 1980)^			
ACh	SC: ↑ ^(Schechtmann et al., 2008)^			
SP	SC: ↑ ^(Linderoth et al., 1992)^ PAG: ↔ ^(Stiller et al., 1995)^			

Ascending nociception is modulated by local segmental mechanisms as well as descending antinociceptive pathways that are reflected by changes in neurotransmitters in the spinal cord dorsal horn. The location of tissue sampling in response to SCS is vitally important as neural circuitry may exhibit a pro- or antinociceptive response to a particular neurotransmitter depending on the site. P-SCS modulates nociceptive signal propagation through a change in the balance of excitatory and inhibitory neurotransmitters, most notably 5-HT and GABA. Little is known about neurotransmitter modulation with B-SCS, HF-SCS and DRG-S. Further work will be necessary to clarify the analgesic mechanisms of these newer paradigms. ‘Up’ and ‘down’ arrows represent increases or decreases, respectively in detection. ‘Sideways’ arrows represent no significant change. Glu (Glutamate), 5-HT (5-hydroxytryptamine, serotonin), GABA (Gamma-aminobutyric Acid), NE (Norepinephrine), ACh (Acetylcholine), SP (Substance P), SC (Spinal Cord), PAG (Periaqueductal Gray), RVM (Rostral Ventromedial Medulla), CSF (Cerebrospinal Fluid)

**Table 2:** Spinal cord stimulation exerts analgesia by activating specific receptor subtypes

Analgesia-Response To:					
Neurotransmitter	Receptor Subtype	P-SCS	B-SCS	HF-SCS	DRG-S
5-HT	5-HT_1_: 5-HT_2_: 5-HT_3_: 5-HT_4_: 5-HT_6_: 5-HT_7_:	↔ ^(Song et al., 2011)^ ↑ ^(Barchini et al., 2012)^ ↑ ^(Song et al., 2011; Barchini et al., 2012)^ ↑↑ ^(Song et al., 2011)^ ↑ ^(Song et al., 2011)^ ↔ ^(Song et al., 2011)^ ↔ ^(Song et al., 2011)^			
GABA	GABA_A_: GABA_B_:	↑ ^(Duggan & Foong, 1985; Song et al., 2011; Barchini et al., 2012)^ ↔ ^(Cui et al., 1996)^ ↑↑ ^(Song et al., 2009; Song et al., 2011; Cui et al., 1996; Barchini et al., 2012)^			
NE	α1 adrenergic: α2 adrenergic: β1 adrenergic: β2 adrenergic:	↑ ^(Barchini et al., 2012)^ ↑↑ ^(Barchini et al., 2012; Schechtmann et al., 2004)^ ↑ ^(Barchini et al., 2012)^ ↑ ^(Barchini et al., 2012)^			
Dopamine	D2 D3	↑ ^(Barchini et al., 2012)^ ↑ ^(Barchini et al., 2012)^			
ACh	M1 mAChR: M2 mAChR: M3 mAChR: M4 mAChR: nAChR:	↑ ^(Schechtmann et al., 2008)^ ↑ ^(Schechtmann et al., 2008; Song et al., 2008)^ ↔ ^(Schechtmann et al., 2008)^ ↑↑ ^(Schechtmann et al., 2008; Song et al., 2008)^ ↔ ^(Song et al., 2009)^ ↔ ^(Schechtmann et al., 2008)^			

Activation of specific receptor subtypes contributes to P-SCS mediated analgesia. Experiments used receptor antagonists and agonists to clarify the role of particular receptors to SCS-mediated analgesia. 5-HT_3_ receptor, GABA_B_ receptor, α2 adrenergic receptor and M4 mAChR pathways were found to contribute to this analgesia to a greater extent than other receptor subtypes. An “up” arrow indicates that activation of that particular receptor subtype contributed to analgesia. ‘Double up’ arrows represent a greater increase and the predominant pathway. ‘Sideways’ arrows represent no contribution to analgesia. Acronyms: Glu (Glutamate), 5-HT (5-hydroxytryptamine, serotonin), GABA (Gamma-aminobutyric Acid), NE (Norepinephrine), ACh (Acetylcholine), 5-HT_1_ (5-hydroxytryptamine receptor 1), 5-HT_2_ (5-hydroxytryptamine receptor 2), 5-HT_3_ (5-hydroxytryptamine receptor 3), 5-HT_4_ (5-hydroxytryptamine receptor 4), 5-HT_6_ (5-hydroxytryptamine receptor 6), 5-HT_7_ (5-hydroxytryptamine receptor 7), GABA_A_ (Gamma-aminobutyric Acid A Receptor), GABA_B_ (Gamma-aminobutyric Acid B Receptor), α1 (Alpha 1 Adrenergic Receptor), α2 (Alpha 2 Adrenergic Receptor), β1 (Beta 1 Adrenergic Receptor), β2 (Beta 2 Adrenergic Receptor), D1 (Dopamine 1 Receptor), D2 (Dopamine 2 Receptor), M1 mAChR (Muscarinic 1 Acetylcholine Receptor), M2 mAChR (Muscarinic 2 Acetylcholine Receptor), M3 mAChR (Muscarinic 3 Acetylcholine Receptor), M4 mAChR (Muscarinic 4 Acetylcholine Receptor), nAChR (Nicotinic Acetylcholine Receptor)

### Gamma-aminobutyric acid

The principle inhibitory neurotransmitter of the CNS, GABA binds to two known GABA receptor classes: GABA_A_ and GABA_B_ (Olsen & DeLorey, 1999). The postsynaptic GABA_A_ receptor complex consists of a multi-target binding domain linked with a chloride ionophore (Olsen & DeLorey, 1999). GABA_A_ receptor agonism increases inward Cl^−^ current, leading to postsynaptic hyperpolarization and increases the firing threshold (Olsen & DeLorey, 1999). The metabotropic postsynaptic GABA_B_ receptor indirectly opens potassium channels though a G-protein-coupled mechanism, leading to membrane hyperpolarization and a similar increase in firing threshold (Olsen & DeLorey, 1999). Uniquely, presynaptic GABA_B_ receptors inhibit neurotransmitter release via a Ca^2+^ dependent mechanism and are critical in presynaptic inhibition (Todd, 2010; Olsen & DeLorey, 1999). Receiving projections from Aβ, Aδ and C fibers, GABAergic interneurons populate laminae I-III and demonstrate GABA positive terminals synapsing on PN (Todd, 2010; Zhang et al., 2014; Sivilotti & Woolf, 1994; Lekan & Carlton, 1995). Of all DH neurons, GABAergic signaling is present in 25% of lamina I, 30% of lamina II and 40% of lamina III (Todd, 2010). Dysfunction of the spinal GABA circuitry in addition to increased excitatory neurotransmitter release is correlated with WDR neuron hyperexcitability (Fig. 4) (Cui et al., 1997; Stiller et al., 1996). Yakhnitsa et al. (1999) demonstrated P-SCS decreases WDR neuron hyperexcitability, noted by depressed evoked potentials and decreased spontaneous discharge on these neurons (Yakhnitsa et al., 1999). This P-SCS-induced suppression of WDR neurons is thought to be due to an increase in DH GABA and concurrent decrease in excitatory glutamate (Cui et al., 1997). SCS modulation of GABA in the DH and other CNS locations is a well-defined phenomenon (Table 1). Contributing mechanisms of analgesia utilizing specific GABA_A_ and GABA_B_ receptor pathways have been clarified using agonist-antagonist experiments (Table 2). Converging lines of evidence confirm that P-SCS employs a GABAergic mechanism in the DH, as evidenced by inhibition of PN firing in response to P-SCS treatment (Sivilotti & Woolf, 1994; Duggan & Foong, 1985). Specifically, administration of a GABA_A_ receptor antagonist reversed PN inhibition produced by P-SCS, demonstrating the importance of DH GABA to P-SCS mediated antinociception (Sivilotti & Woolf, 1994; Duggan & Foong, 1985). Notably, P-SCS responders in an allodynic rat model were observed to have increased levels of GABA in the dorsal horn, whereas non-responders and sham animals exhibited no change, indicating that P-SCS mediated DH GABA release may prevent allodynia (Stiller et al., 1996). At the segmental level, suppression of glutamate release is dependent on presynaptic activation of GABA_B_ receptors, which is likely more important to the P-SCS interneuron-mediated inhibition of PN firing than activation of postsynaptic GABA_A_ receptors (Cui et al., 1997; Stiller et al., 1996; Cui et al., 1996). Expanding on these findings, Cui et al. (1997) found that administration of an intrathecal GABA_B_ receptor agonist would transform P-SCS non-responding animals into responders (Cui et al., 1997). This finding is similar to the work by Song et al. (2011) that shows greater analgesia with 5-HT_3_ receptor-mediated GABA_B_ signaling than GABA_A_ signaling (Song et al., 2011). It remains unclear which GABA-mediated effects are due to local interneuron circuitry and which are the result of activation of the serotonergic DAS. As noted previously, P-SCS recruits the serotonergic DAS, which employs downstream GABAergic mechanisms of antinociception. Using P-SCS and implanted microdialysis catheters, Stiller et al. (1995, 1996) and Linderoth et al. (1993) demonstrated an increase in extracellular GABA in the DH and decreased levels in the PAG (Stiller et al., 1996; Linderoth et al., 1993; Stiller et al., 1995). This decrease in PAG GABA argues that P-SCS relieves inhibition of the serotonergic DAS, which relays through the RVM, allowing the system to exert descending control over DH PN.

In clinical studies, Lind et al. (2004, 2008) showed that administration of an intrathecal GABA_B_ receptor agonist (Baclofen) significantly enhanced the analgesia of P-SCS and rescued non-responders, echoing results of preclinical animal models (Lind et al., 2008; Lind et al., 2004). Cortical and subcortical pain circuits may also be modulated by P-SCS utilizing a GABAergic mechanism. Moens et al. (2013) studied 20 FBSS patients who were treated with P-SCS and underwent functional magnetic resonance imaging (fMRI) 7–10 days post-implantation (Moens et al., 2013). They discovered increased GABA and decreased glucose signals in the ipsilateral thalamus, potentially explained by orthodromic activation of the paleospinothalamic pathway. They hypothesized that projections from the reticular formations to GABAergic nuclei in the thalamus, hypothalamus and limbic system may indicate an interference with the affective component of pain (Moens et al., 2013; Moens et al., 2012). This would represent an additional mechanism of P-SCS efficacy. In summary, P-SCS mediated GABAergic mechanisms have been described at three targets. First, P-SCS activates GABAergic inhibitory interneurons at the dorsal horn, either directly or by recruiting the serotonergic DAS. Second, P-SCS results in decreased GABAergic signaling in the PAG, which results in disinhibition and thus activation of the serotonergic DAS. Lastly, P-SCS orthodromically activates thalamic GABAergic neurons, which may modulate cortical processing and thalamocortical dysrhythmia. Converging lines of evidence derived from preclinical and emerging clinical work suggest a central role for GABAergic P-SCS mediated analgesia. These GABAergic pathways likely contribute to the observed clinical analgesic effects of P-SCS at both the segmental and supraspinal levels. Clearly, more work is needed to clarify these pathways in emerging paradigms, including DRG-S and PF-SCS (Tables 1 and 2).

### Norepinephrine

Norepinephrine (NE), often referred to as Noradrenaline, is a catecholamine neurotransmitter produced primarily in the LC and released in the spinal cord DH (Hayashida et al., 2008a). In neuropathic pain, not only is there an increase in DH NE release and adrenergic axon sprouting, but corroborative evidence suggests an inability to recruit the DAS may contribute to chronic pain states (Hayashida et al., 2008b; Witting et al., 2003). NE has antinociceptive effects through presynaptic inhibition of primary Aδ and C fibers, postsynaptic inhibition of WDR and NS neurons, and activation of inhibitory interneurons (Hayashida et al., 2007; Pertovaara, 2006). In a rat model, the activation of inhibitory interneurons by NE increases GABAergic and glycinergic postsynaptic currents as measured in the SG (Baba et al., 2000a; Baba et al., 2000b). While this supports the segmental role for NE-mediated analgesia in the DH, converging lines of evidence now suggest that local DH NE release is unlikely to be augmented by P-SCS (Tazawa et al., 2015; Song et al., 2013b). Song et al. demonstrated that while the LC is activated in a neuropathic rat model treated with P-SCS, no change was observed in DH NE (Song et al., 2013b). Tazawa et al. (2015) supported this conclusion and further clarified that no increase in DH NE was observed with P-SCS (Tazawa et al., 2015). However, scant clinical reports confirmed concentrations of NE increased in CSF sampled after P-SCS therapy. Two separate human studies to date show an immediate increase in CSF NE concentration pre-to-post P-SCS (Levin & Hubschmann, 1980; Liu et al., 2008). However, while Levin et al. (1980) showed an immediate increase in CSF NE, concentrations returned to baseline after 5 min, calling into question the durable role of the noradrenergic DAS in P-SCS. In total, the current literature suggests that rather than recruiting descending NE fibers, *orthodromic activation of the LC* by P-SCS likely relays through the PAG, in essence reinforcing descending antinociception through the serotonergic DAS previously discussed (Fig. 5). The clinical longevity and significance of potential increases in CSF NE mediated by P-SCS, PF-SCS, and DRG-S remains to be determined.

### Acetylcholine

Though the mechanism is not clearly defined, cholinergic inputs to PN play a role in modulating nociceptive signals via spinal interneuron circuitry (Foreman, 2012). Clonidine, a presynaptic alpha 2 (α_2_) adrenergic receptor agonist, exerts its analgesic effect largely through a cholinergic mechanism at the spinal level (Foreman, 2012). After discovering that clonidine may potentiate the analgesic effect of P-SCS in a rat model, Schechtmann et al. (2008) demonstrated lower basal DH ACh in nerve-lesioned animals and an increase in DH ACh in P-SCS responding animals (Schechtmann et al., 2008). Using agonist-antagonist studies, their group further noted reversal of analgesia with administration of selective and non-selective muscarinic acetylcholine receptor (mAChR) antagonists (Table 2). Specifically, they noted that muscarinic 1 (M1) mAChR and M4 mAChR contributed to P-SCS mediated analgesia while nicotinic and muscarinic 3 (M3) mAChrR antagonism had no effect on PWT. Supporting this finding and further clarifying the receptor subtypes involved, Song et al. (2008) found that intrathecal muscarinic 2 (M2) mAChR and M4 mAChR agonists reversed P-SCS non-responders (Song et al., 2008). However as noted above, Song et al. (2009) showed that 5-HT signaling in the serotonergic DAS did not involve the M4 mAChR pathway (Song et al., 2009). Thus, cholinergic signaling in P-SCS may represent a novel, independent mechanism exclusive of the serotonergic DAS or a parallel, redundant pathway. In a subsequent randomized clinical trial, Schechtmann et al. (2010) delivered sub-analgesic doses of intrathecal clonidine or baclofen combined with P-SCS in non-responding patients with prior P-SCS devices. Similar to the work by Lind et al. (2004, 2008) (Lind et al., 2008; Lind et al., 2004), Schechtmann et al. (2010) showed intrathecal clonidine significantly improved pain scores (in two patients) when combined with P-SCS. Together, this provides some clinical evidence for cholinergic augmentation in P-SCS non-responders (Schechtmann et al., 2010). In sum, cholinergic mechanisms play a role in P-SCS mediated segmental antinociception. However, as with other neurotransmitters, work is needed to clarify the clinical contribution of cholinergic mechanisms to the analgesic effects of newer paradigms, including PF-SCS and DRG-S.

### Endogenous opioids

Endogenous peptides of the brain and spinal cord, β-endorphins, enkephalins and dynorphins are ligand classes that activate μ, κ and δ-opioid receptors. These ligands exert analgesia segmentally through direct receptor activation in addition to recruitment of the serotonergic and noradrenergic DAS (Benzon et al., 2014). A well-known opioidergic center, the PAG communicates with the DAS, medulla, dorsal raphe nucleus and the LC (Benzon et al., 2014). Causing hyperpolarization and signal inhibition through a G-protein coupled mechanism, μ, κ and δ-opioid receptors are widely present on pre and post-synaptic neurons of the DH (Benzon et al., 2014). As the μ-opioid receptor is known to play a role in the development of windup, modulation of the endogenous opioidergic system may potentially explain analgesia with SCS treatment (Guan et al., 2006). Providing a physiologic basis for this hypothesis, Wang et al. (2003) has shown that activation of the serotonergic DAS increases enkephalin and dynorphins in the DH (Wang et al., 2003). In line with this finding, Ding et al. (2008) showed that P-SCS resulted in an increase in thoracic DH dynorphin in a rat angina model (Ding et al., 2008). This suggests the possibility that κ opioid receptor activation may contribute to the analgesic effects of P-SCS (Ding et al., 2008). In a subsequent SNI rat model, Sato et al. (2013) observed improved PWT after frequency-specific P-SCS at 4 Hz and 60 Hz. Notably, they observed that the improvement with 4 Hz and 60 Hz was reversed upon administration of naloxone and naltrindole, respectively (Sato et al., 2013). Similar to what has been observed with transcutaneous electrical nerve stimulation (TENS), this finding indicates frequency-specific activation of μ and δ-opioid receptors with P-SCS (Sato et al., 2013; Chandran & Sluka, 2003). Despite frequency-specific activation of opioidergic pathways, the role of this mechanism in P-SCS is likely temporally limited to the first few days of stimulation. In an SNI model treated with P-SCS at 3 and 7 days, naloxone administration attenuated early but not late reversal of hyperalgesia (Sun et al., 2017). Postulating that the temporal reduction in efficacy occurred through endogenous opioid tolerance, Chandran et al. (2002), observed similar findings in patients treated with both low and high frequency TENS (Chandran & Sluka, 2003). Limited evidence from clinical studies appears to mirror the results of these preclinical investigations. In 17 patients with chronic pain admitted to the neurosurgical service, Tonelli et al. (1988) implanted and initiated single lead P-SCS during hospital admission, sampling their CSF one to two days prior and again one day after implantation and initiation of therapy (Tonelli et al., 1988). Responders were noted to have a significant increase in CSF β-endorphin and β-lipotropin, a prohormone of the β-endorphin peptide. Unfortunately, CSF was not sampled at any additional timepoints to clarify whether this increase in β-endorphin was sustained (Tonelli et al., 1988). In other clinical work, Freeman et al. (1983) seemed to agree with preclinical models regarding the lack of sustained opioidergic mechanisms with P-SCS (Freeman et al., 1983). Specifically, naloxone did not reverse analgesia from P-SCS after 30 days of stimulation in patients having relief with TENS or P-SCS. This continues to suggest that recruited opioidergic mechanisms may contribute to the initial analgesic efficacy of P-SCS (during P-SCS trial and subsequent implant) but the longevity of this mechanism is questionable. There is likely an analgesic ceiling effect in regard to opioidergic pathways recruited by P-SCS. In a rat SNI model treated with P-SCS, administration of the opioidergic enhancer proglumide had no additive or synergistic effect on PWT or physical activity levels (Inoue et al., 2017). Tolerance and time-limited opioidergic analgesia that contribute to the clinical efficacy of P-SCS may also apply to PF-SCS. To date, one study of non-lesioned rat lumbar spinal cord slices demonstrated frequency-dependent opioid release from DH neurons, with a maximal release at 500 Hz (Song & Marvizón, 2003). Further work is needed to determine the role and longevity of opioidergic mechanisms in all modes of SCS. Moreover, additional studies are needed to evaluate frequency-specific endogenous opioid release in P-SCS and PF-SCS and whether these mechanisms are predominantly segmental or supraspinal.

## SCS modulates Neuroinflammatory pain regulation

### Signatures of glial activation

**Fig. 6:**
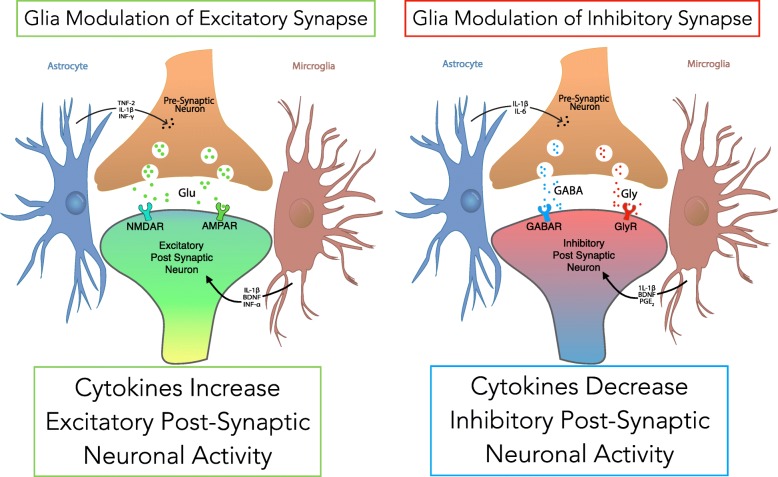
Local Glia Modulate Synaptic Transmission After Nerve Injury. Glia are intimately associated with spinal excitatory and inhibitory synapses. The release of cytokines and glial mediators on pre and post-synaptic terminals modulates the activity of that synapse. Cytokines released at glutaminergic excitatory synapses augment transmission while cytokines released at GABAergic and Glycinergic synapses attenuates the signal. The net effect of gliosis and release of glial mediators is an increase in spinal cord pain transmission. Novel modes of SCS may modulate glial activity, thereby enacting their antinociceptive mechanisms through this pathway. Acronyms: Glu (Glutamate), GABA (Gamma Aminobutyric Acid), Gly (Glycine), TNF-α (Tumor Necrosis Factor Alpha), IL-1β (Interleukin 1β), IFN-γ (Interferon Gamma), NMDAR (NMDA Receptor), AMPAR (AMPA Receptor), BDNF (Brain Derived Neurotrophic Factor), GABAR (GABA Receptor), GlyR (Glycine Receptor), PGE_2_ (Prostaglandin E_2_)

Critical to the structure, metabolism and immunity of both the CNS and peripheral nervous system (PNS), glia are non-neuronal cells intimately associated with neurons. Glia of the CNS include microglia, astrocytes, oligodendrocytes and ependymal cells while those of the PNS include satellite glial cells (SGCs) and Schwann cells (Ji et al., 2013). It is now understood that glia also provide a functional microenvironment modulating signal transduction, neuroplasticity and synaptic pruning (Tremblay et al., 2011). In response to nerve injury, striking changes are seen in glial morphology, concentration, cellular signaling, receptor regulation and mediator release. Termed gliosis, these responses together constitute a phenotypic transformation that alters signaling by changing the synaptic neuron-glia mediator balance (Fig. 6) (Ji et al., 2013). Temporally correlating with the onset of neuropathic pain, PNS and CNS insults including nerve injury and cord hypoxemia lead to reactive gliosis. Specifically, reactive gliosis leads to increased post-synaptic potentials at excitatory synapses and decreased post-synaptic potentials at inhibitory synapses (Fig. 6). Through complex signaling mechanisms, these changes result in increased PN firing and pathologic nociception (Ji et al., 2013). While microglial activation occurs 24 h after injury and is limited to 3 months, astrocyte activation occurs 3 days after injury and is maintained (Coyle, 1998; Ledeboer et al., 2005; Mika, 2008; Mika et al., 2009). Activation and intracellular signaling culminate with the release of glial mediators, which exhibit their downstream effect on pre and post-synaptic targets (Fig. 6) (Ji et al., 2013; Mika et al., 2013). Mediator classes known to influence nociceptive transmission include nitric oxide (NO), cytokines, chemokines, complement components and other bioactive factors, some of which have been examined in response to SCS (Table 3) (Mika et al., 2013). Given that glial activation is important to the development and maintenance of neuropathic pain and that glial inhibition improves mechanical allodynia, one potential mechanism of SCS is glial modulation (Watkins et al., 2001a; Watkins et al., 2001b). In a seminal pre-clinical study, Sato et al. (2014) showed decreased glial activation after P-SCS therapy correlating with improved PWT (Sato et al., 2014). Specifically, P-SCS decreased immunostaining of microglia marker OX-42 and astrocyte markers GFAP and MCP-1 in the superficial and deep DH lamina (Sato et al., 2014). Changes in glial activation are also observed at the DRG.

**Table 3:** Evidence for the response of cytokines and neurotrophic factors to spinal cord stimulation (SCS) paradigms

			Tissue Sampled and Measured Change with SCS			
Neuromediator	WDR/NS Firing	Nociceptive Effect	P-SCS	B-SCS	HF-SCS	DRG-S
Measured Centrally:						
BDNF	Increased	Algesia	DRG: ↔ ^(Tilley et al., 2017)^			
C3	Increased	Algesia	SC: ↑ ^(Stephens et al., 2018)^			
c-Fos	Increased	Algesia	SC: ↑ ^(Tilley et al., 2016)^			
IL-1β	Increased	Algesia	SC: ↑ ^(Stephens et al., 2018; Vallejo et al., 2016)^ DM: ↑ ^(Tilley et al., 2019)^ DRG: ↑ ^(Tilley et al., 2017)^			
IL-6	Increased	Algesia	DRG: ↓ ^(Tilley et al., 2017)^			
TNF-a	Increased	Algesia	SC: ↑ ^(Tilley et al., 2016)^			
VEGF	Increased	Algesia	CSF: ↓ ^(McCarthy et al., 2013)^			
CXCL16	Decreased	Analgesia	SC: ↑ ^(Vallejo et al., 2016)^ DRG: ↑ ^(Vallejo et al., 2016)^			
GDNF	Decreased	Analgesia	CSF: ↑ ^(McCarthy & McCrory, 2014a)^			
IL-1α	Decreased	Analgesia	SC: ↑ ^(Vallejo et al., 2016)^			
IL-10	Decreased	Analgesia	DM: ↑ ^(Tilley et al., 2019)^			
NpY	Decreased	Analgesia	DRG: ↔ ^(Tilley et al., 2017)^			
VIP	Decreased	Analgesia	DRG: ↔ ^(Tilley et al., 2017)^			
Measured Peripherally						
CXCL10	Increased	Algesia	IF: ↓ ^(Kriek et al., 2018)^			
IFN-γ	Increased	Algesia	IF: ↓^(Kriek et al., 2018)^ Plasma: ↔^(Kamieniak et al., 2019a)^			
IL-1β	Increased	Algesia	Plasma: ↔^(Kamieniak et al., 2019a)^			
IL-2	Increased	Algesia	IF: ↓^(Kriek et al., 2018)^			
IL-6	Increased	Algesia	IF: ↔^(Kriek et al., 2018)^ Plasma: ↔^(Kamieniak et al., 2019a)^			
IL-12	Increased	Algesia	IF: ↓^(Kriek et al., 2018)^			
IL-15	Increased	Algesia	IF: ↓ ^(Kriek et al., 2018)^			
TNF-a	Increased	Algesia	IF: ↔^(Kriek et al., 2018)^ Plasma: ↔^(Kamieniak et al., 2019a)^			
VEGF	Increased	Algesia	IF: ↓ ^(Kriek et al., 2018)^			
IL-4	Decreased	Analgesia	IF: ↓^(Kriek et al., 2018)^			
IL-5	Decreased	Analgesia	IF: ↓^(Kriek et al., 2018)^			
IL-10	Decreased	Analgesia	IF: ↓^(Kriek et al., 2018)^ Plasma: ↔^(Kamieniak et al., 2019a)^	Plasma: ↑^(Kinfe et al., 2017)^		
TGF-β	Decreased	Analgesia	Plasma: ↔^(Kamieniak et al., 2019a)^			

Measured centrally or peripherally, changes in neuromediator concentration and gene expression have been observed with P-SCS, sampled from multiple tissues. As glia are intimately associated with neurons and nociceptive signal propagation, small changes in local and systemic neuromediators can affect glial activation, recruitment and propagation of the inflammatory cascade. While evidence suggests that P-SCS exerts antinociceptive efficacy partially through modulation of glia, inflammation and gene expression, little is known about the mechanisms of other stimulation modalities including B-SCS, HF-SCS and DRG-S. More work is necessary to elucidate the analgesic mechanisms of theses stimulation paradigms. Up’ and ‘down’ arrows represent increases or decreases, respectively, in concentration and expression. ‘Sideways’ arrows represent no significant difference. Acronyms: BDNF (Brain-Derived Neurotrophic Factor), C3 (Complement Component 3), c-Fos (proto-oncogene), IL-1α (Interleukin 1 Alpha), IL-1β (Interleukin 1 Beta), IL-2 (Interleukin 2), IL-6 (Interleukin 6), IL-10 (Interleukin 10), IL-12 (Interleukin 12), IL-15 (Interleukin 15), TNF-α (Tumor Necrosis Factor Alpha), VEGF (Vascular Endothelial Growth Factor), CXCL16 (Chemokine C-X-C Motif Ligand 16), GDNF (Glial Cell Line-Derived Neurotrophic Factor), NpY (Neuropeptide Y), VIP (Vasoactive Intestinal Peptide), IFN-*γ* (Interferon Gamma), TGF-β (Transforming Growth Factor Beta), DRG (Dorsal Root Ganglion), SC (Spinal Cord), DM (Dura Mater), CSF (Cerebrospinal Fluid), IF (Interstitial Fluid)

DRG SGCs are perfectly positioned within the sandwich synapse to regulate nociceptive transmission (Todd, 2010; Rozanski et al., 2013). Encircling a single neuron with a thin sheath intrasynaptically, SGCs are implicated in nociceptive modulation (Hanani, 2005). Having multiple bioactive receptors, SGC modulate neural activity through the release of mediators that have been shown to alter neural activity via the P2X3 pathway. The upregulation of this pathway promotes abnormal nociception in rats (Hanani, 2005; Chen et al., 2008). In a L5 SNI model, SGC activation was observed while glial inhibitors administered to the ipsilateral DRG provided alleviation of mechanical allodynia (Liu et al., 2012a). Moreover, SGC reorganization and the formation of new neural contacts and gap junctions has been observed after peripheral nerve axotomy (Hanani et al., 2002). Given the proximity to the DRG, DRG-S may modulate SGC activation. In a tibial nerve injury model, Pan et al. (2016) first showed that DRG-S normalized glial fibrillary acidic protein (GFAP) and activating transcription factor 3 (ATF-3), markers of astrocytic activation and neuronal injury (Pan et al., 2016). Moreover DRG-S was noted to reverse cold and mechanical hypersensitivity, suggesting that DRG-S impacts SGC-mediated neuroinflammation (Pan et al., 2016). While evidence is still limited, SGCs clearly play a role in the development of pain states, modulation of which may explain the efficacy of DRG-S. It is unknown if other SCS paradigms modulate SGC activity or are efficacious at the DRG. To date, no human clinical work has yet been completed that characterizes these promising observations seen in DRG-S treated preclinical models. Taken together, neuroinflammatory glial mechanisms are critical in chronic pain maintenance while glial-mediated mechanisms of P-SCS, PF-SCS and DRG-S remain to be elucidated. While a comprehensive examination of glial activation is beyond the scope of this review, the evidence for glial mediator response to modes of SCS are carefully summarized in Table 3 and reviewed below.

### Cytokines, neurotrophic factors and biomarkers

Critical to the induction and maintenance of chronic pain, dysregulation of local cytokine and neurotrophic factor signaling directly and indirectly influences AP generation in the PN. The release of inflammatory cytokines, neurotrophic factors and other mediators operates under a positive feedback mechanism of autocrine and paracrine regulation. While likely evolutionarily advantageous in recruiting the inflammatory cascade, gliosis and the resultant local inflammatory response becomes a maladaptive and injurious process in chronic pain. While evidence for glial modulation in response to SCS is mounting, the effect of SCS on the local and systemic inflammatory responses remains unclear.

Upregulated in gliosis after nerve injury, the proinflammatory cytokines interleukin-1β (IL-1β), IL-6, IL-8 and TNF-α exert potent pronociceptive action (Bjurstrom et al., 2016). Of particular importance, IL-1β and TNF-α increase PN excitability, reinforce glial activation and further recruit the inflammatory response (Bjurstrom et al., 2016). Concentrations of CSF proinflammatory cytokines are increased in multiple chronic pain states including osteoarthritis, CRPS, postherpetic neuralgia and fibromyalgia (Bjurstrom et al., 2016). Patients with indications for SCS therapy including chronic low back pain, lumbar radiculopathy and failed back surgery syndrome (FBSS) were also noted to have elevations in proinflammatory cytokines (Bjurstrom et al., 2016). Post P-SCS effects in pre-clinical models have been evaluated with regard to cytokine levels, summarized in Table 3. In non-nerve injury models treated with P-SCS, Tilley et al. (2009) demonstrated increased expression of meningeal TNF-α and IL-10, but no change was noted in IL-6 or IL-1β (Tilley et al., 2009). Later, Tilley et al. carefully separated the meninges into its three layers and measured cytokine expression following P-SCS (Tilley et al., 2019). They found increases in IL-1β and IL-10 within the dura mater, correlated in a dose-dependent manner to the delivered current (Tilley et al., 2019). Further, the concentration of IL-6, a pleiotropic cytokine, was notably related to delivered current with a bell-shaped relationship (Tilley et al., 2019). Together, these studies demonstrate a possible relationship between cytokine expression and delivered current. In a SNI rat model treated with P-SCS, Tilley et al. (2017) demonstrated decreased DRG expression of IL-6, which is normally upregulated in neuropathic pain models (Tilley et al., 2017; Ha et al., 2001) and they also noted increased expression of the IL-1β (Tilley et al., 2017). Evaluation of clinical cytokine profiles in SCS therapy may not only elucidate a potential mechanism, but also serve to guide clinical therapy and response. Measuring interstitial fluid sampled from artificial skin blisters, Kriek et al. (2017) investigated the immunomodulatory effects of P-SCS on CRPS (Kriek et al., 2018). They showed significant reduction in interferon-γ-inducible protein 10 and vascular endothelial growth factor (VEGF). However, they ascertained no significant change in the proinflammatory cytokines IL-2, IL-6, IL-12, IL-15, IL-17, IFN-γ, or TNF-α or the anti-inflammatory cytokines IL-4, IL-5, IL-10 or IL-13 (Table 3) (Kriek et al., 2018). In clinical work by Kinfe et al. (2017) evaluating the effects of B-SCS, peripheral blood concentrations of the anti-inflammatory cytokine IL-10 interestingly increased in responders, however, changes in levels of other cytokines were not determined to be statistically significant (Kinfe et al., 2017). This finding has to be carefully considered, as other variables including reduced pain levels, changes in mood or improvement in sleep could also explain the finding. The plausibility that an electrical pulse applied to the thoracic spinal cord could directly modulate systemic cytokine levels is difficult to rationalize. As there are no published clinical studies evaluating CSF cytokine levels after treatment with P-SCS, further work is clearly needed to determine if cytokine modulation is a contributing mechanism to analgesia.

Nerve Growth Factor (NGF), Brain Derived Neurotrophic Factor (BDNF) and Glial Cell Line-Derived Neurotrophic Factor (GDNF) are essential neurotrophic factors for maintenance and regeneration after PNS or CNS injury. Known to activate and sensitize nociceptive neurons, inflammation triggers NGF expression in mast cells, macrophages, and Schwann cells (Pezet & McMahon, 2006). CSF neurotrophic factor concentrations are increased in patients with chronic lower back pain, lumbar radiculopathy and failed back surgery syndrome, all known targets of SCS therapy (Pezet & McMahon, 2006). Similarly, increased BDNF expression is observed in DRG neurons, microglia, and astrocytes in inflammatory and neuropathic pain models (Ha et al., 2001; Pezet & McMahon, 2006; Vanelderen et al., 2010; Biggs et al., 2010). GDNF, expressed in astrocytes, peripheral tissues and active chondrocytes, is thought to contribute to neuroinflammatory mediated pain (Bjurstrom et al., 2016; Pezet & McMahon, 2006). In 2017, Tilley and colleagues showed increased expression of BDNF after SNI, though no change was seen with application of P-SCS (Tilley et al., 2017). In two separate studies of FBSS patients being treated with P-SCS by McCarthy et al. (2013, 2014), CSF concentrations of the neurotrophic factors BDNF and GDNF were observed to be high in P-SCS treated patients compared to healthy controls (McCarthy et al., 2013; McCarthy & McCrory, 2014b). However, they did not observe any effects of P-SCS therapy on BDNF and GDNF levels. Other mediators play a role in nociception and provide a possible contributing mechanism to the efficacy of SCS. In FBSS patients treated with P-SCS, McCarthy et al. (2013) described elevated CSF concentration of the inflammatory chemokine monocyte chemoattractant protein 1 (MCP-1) (McCarthy et al., 2013). This remains the only published report of an elevated chemokine with P-SCS treatment. Significant changes have also been observed in neuroimmune and nociceptive signaling proteins after treatment with P-SCS, measured in the CSF by proteomic mass spectrometry (Lind et al., 2016). Lastly, one study evaluated the CSF concentrations of non-ionized periodic elements in patients with implanted P-SCS devices (Korvela et al., 2016). While no change was noted in any element before or after P-SCS therapy, significantly higher concentrations of several elements were noted in patients with chronic pain compared to healthy controls (Korvela et al., 2016). However, it is difficult to interpret the lack of change with P-SCS therapy as these patients potentially had an inadequate washout period.

Although there is a paucity of clinical work, some preclinical studies support the effect of P-SCS on biomarkers of chronic pain. Collectively, there is evidence that P-SCS modulates neuroinflammation and nociception (Table 3). Variability in the published literature and gaps in knowledge currently prevent the clinician from identifying the response of neuroinflammation to SCS. While Table 3 summarizes the available preclinical and clinical data, it is difficult to make definitive statements regarding the response of cytokines and other mediators to SCS therapy. Although the current literature is sparse and divided, pre-clinical work is underway that may clarify the response of these complex inflammatory signaling cascades to multiple modes of SCS (Tilley et al., 2019). As such, there is a clear need for this pre-clinical work to translate to clinical studies that carefully measure immunomodulatory profiles, both centrally and peripherally.

### Sexual dimorphism, inflammation and potential mechanisms in SCS

Evidence has emerged for the existence of sex-specific differences in chronic pain pathways (Fillingim et al., 2009). In addition to a higher incidence of neuropathic pain, musculoskeletal pain, fibromyalgia, low back pain and migraine, women are also noted to exhibit increased sensitivity and decreased tolerance to applied experimental pain stimuli (Fillingim et al., 2009). Though the etiology of these differences is likely multifactorial, investigative efforts have begun to unveil sex-specific mechanisms of hyperalgesia and allodynia in preclinical models. While it is unknown if there is a sex-based discrepancy in response to SCS, there exists a potential for sex-specific SCS device programming or pharmacotherapeutic adjuvants to augment clinical benefit. Thus, further elucidation of these pathways may translate to the clinical use of targeted therapies for both male and female patients.

In order to limit experimental variability, the majority of animal models used to construct the foundations of preclinical pain research have been male. Sorge et al. (2011) first identified a male-specific, testosterone-dependent pathway involving spinal toll-like receptor 4 (TLR4), a receptor primarily present on spinal microglia (Sorge et al., 2011). Their work suggested that a TLR4-independent pathway was responsible for allodynia in females. Interestingly, the administration of testosterone to female mice acted as a ‘switch’, activating the male-specific pathway (Sorge et al., 2011). In their seminal work published in 2015, Sorge and Mogil further characterized this observed sexual dimorphism, noting both conserved and unique pathways to hypersensitivity and allodynia (Sorge et al., 2015). Specifically, while intrathecal NMDA antagonism reversed mechanical hypersensitivity regardless of gender, they noted a male-specific microglial upregulation of the P2X4 receptor (P2X4R), crucial to the development of allodynia via the MAPK-dependent synthesis of BDNF. Their group further used dorsal horn gene expression and tamoxifen-dependent microglial BDNF-knockouts to demonstrate that while mechanical hypersensitivity in males is dependent on microglia, *the female correlate relies on a mechanism of adaptive immunity, likely dependent on T-cells (Sorge et al.,* 2015*)*. With suppression of the adaptive immunity pathway, females revert to the male-specific, microglia-dependent pathway (Sorge et al., 2015). Confirming this observation in another species, their group used SNI and CCI rat models to identify microglia and P2X4R as key points of divergence between the sexes. Moreover, they noted that while intrathecal administration of male P2X4R-stimulated microglia caused allodynia in both male and female naïve rats, injecting female P2X4R-stimulated microglia had no effect on animals of either sex (Mapplebeck et al., 2018). In evaluating the increased expression of P2X4R, the investigators evaluated IRF5, a transcription factor known to regulate P2X4R gene expression. Interestingly, despite elevated spinal levels in both male and female nerve injury models, IRF5 was noted to exclusively bind to the P2X4R gene promoter region in male rats, but not females (Mapplebeck et al., 2018). Together, evidence from these preclinical models suggests that the development of mechanical hypersensitivity may rely on an innate and adaptive immune process in males and females, respectively. If proven in human studies, these sex differences may lead to a poor clinical response to microglial inhibitors in females and account for the current reported lack of efficacy in clinical trials that include both genders (Brings & Zylka, 2015). In line, SCS mechanism that may act through TLR4, P2X4R, or IRF5 pathways may be differentially regulated in male and female patients.

With an increased incidence of chronic pain and autoimmune disease amongst women, it is conceivable that adaptive immunity and T cells link the pathophysiology of these seemingly separate processes. Known to induce allodynia through a T cell mediated mechanism and normally hidden from immune surveillance, a conserved region of myelin basic protein (MBP) is a degradation product of the protective sheath enwrapping Aβ Fibers (Liu et al., 2012b). After demonstrating that the injection of MBP 84–104 fragment into naïve nerves induced an ipsilateral inflammatory and immune cascade, Liu et al. (2012) postulated that nerve injury and repeated exposure of this hidden region on MBP led to a deleterious, allodynia-reinforcing immune reaction (Liu et al., 2012b). Expanding on this, Chernov et al. (2018) interestingly demonstrated that sciatic nerve injection of the MBP 84–104 fragment induced long-lasting mechanical allodynia in female, but not male animals (Chernov et al., 2018). Moreover, they also observed sexual dimorphism in gene expression profiles measured in the sciatic nerve, DRG and spinal cord post injection. Additional work by the same group further supports an autoimmune mechanism in females, demonstrating seropositivity for autoantibodies to the MBP 84–104 fragment (Hullugundi et al., 2017). A proinflammatory protease responsible for the cleavage of MBP to its immunogenic products, matrix metalloproteinase 9 (MMP-9) contributes to acute and late phase peripheral neuropathy (Liu et al., 2012b; Remacle et al., 2018). Interestingly, however MMP-9 activity and disinhibition were comparably elevated in both male and female CCI models (Remacle et al., 2018). Along with their previous work, this argues that the sex-specific pathways diverge at the immune response to MBP. To date there is only one clinical translational P-SCS study that examined MMP-9 response pre-to-post P-SCS, finding no difference in MMP-9 or its inhibitor, though it did not evaluate for differences by gender (Kamieniak et al., 2019b). While sexual dimorphism likely plays a role in the innate and adaptive immune-mediated development of allodynia, it remains unclear if SCS is capable of achieving antinociception via modulation of these pathways.

Sex-specific differences have also been observed in opioid responsiveness and signaling, though this remains disputed (Mogil, 2012). Preclinical research implicates differential activation of microglia and TLR4 at the supraspinal level as responsible for the observed sex-dependent opioid response. In an animal model, Doyle et al. (2017) demonstrated that activation of female microglia in the PAG reduced typical antinociceptive pathways through the TLR4 specific pathway (Doyle et al., 2017). Notably, intra-PAG injection of naloxone, blocking morphine’s interaction with TLR4, increased female analgesia to the level of their male counterparts. As supraspinal mechanisms of SCS intricately engage the PAG and DAS, there is a clear need to further our understanding of the interaction between SCS and sex-specific opioidergic antinociception. Current clinical studies of SCS are predominantly carried out in both genders without sex comparisons. In one recent CSF-sampled proteomic study of 11 female and 3 male patients with P-SCS, Lind et al. (2016) demonstrated that the greatest changes occurred in neuroprotection, synaptic plasticity, nociceptive signaling and immune regulation (Lind et al., 2016). While this predominantly female study did not assess for sexual dimorphism, advanced techniques including protein and gene profiling are capable of identifying the interaction between sex, neuro-immunity and inflammation (Ray et al., 2019). To our knowledge, no preclinical or clinical studies have evaluated the sex-specific response to P-SCS, PF-SCS, DRG-S or ECAP-SCS. Given the emerging understanding of these sexually dimorphic pathways, investigation is warranted to determine if SCS therapies alone or in combination with pharmacotherapy have a differential effect on male or female patients.

## Quantitative sensory testing

Utilized both as a clinical and basic science tool, quantitative sensory testing (QST) evaluates the small and large fibers, which detect changes in temperature as well as vibration and electrical stimulation, respectively (Shealy et al., 1970). Categorically, undergoing QST yields sensory data with regard to a particular stimulus. This includes the threshold, which is the minimum sensory input for the subject to experience the onset of change. Modern QST evaluates thermal, mechanical vibratory and electrical paresthesia thresholds. QST also assesses for tolerance, the point at which the stimulus causes unbearable discomfort. Heat, cold, mechanical and electrical tolerances can be measured by QST. As it evaluates the function of the small and large fibers, QST has contributed to both the elucidation of P-SCS MOA as well as has become a predictive tool for its efficacy. Pioneering the effect of P-SCS on QST, Shealy et al. (1970) first demonstrated an increase in deep muscle pain threshold (Shealy et al., 1970) while Lindblom et al. (1975) reported increased tactile and vibratory thresholds but no change in pinch pressure threshold (Lindblom & Meyerson, 1975). Interestingly, a subsequent group found that electrical thresholds were only increased within the area of paresthesia produced by P-SCS (Doerr et al., 1978). More recently, Mironer et al. (2000) found that P-SCS also increased electrical tolerance, which interestingly correlated with P-SCS mediated pain reduction (Mironer & Somerville, 2000). While diverging evidence from Alo et al. (1999) did not replicate the findings of increased electrical tolerance or correlation with pain reduction from P-SCS, they interestingly noted that P-SCS decreased electrical threshold bilaterally irrespective of pain laterality (Aló & Chado, 2000). While warmth threshold, heat pain threshold and heat pain tolerance were increased in two QST studies post P-SCS, Marchand et al. (1991) further noted no change in visual light threshold or tolerance, arguing against a global mechanism for P-SCS-mediated analgesia and rather for a targeted segmental or supraspinal mechanism (Marchand et al., 1991; Ahmed et al., 2015). Though smaller and older studies seems to demonstrate a clear effect of P-SCS on QST metrics (Münster et al., 2012; Rasche et al., 2006; Burkey & Abla-Yao, 2010; Cata et al., 2004), newer diverging lines of evidence demonstrate lackluster results (Meier et al., 2015; Biurrun Manresa et al., 2015), including the largest controlled study to date (Kemler et al., 2001). While the etiology of the discrepancy remains to be elucidated, a review of published literature is altogether temporally summarized in Table 4. Very limited evidence exists for the effect of novel SCS paradigms on QST metrics. To date the only clinical QST study comparing HF-SCS to P-SCS, Youn et al. (2015) demonstrated that HF-SCS alters mechanical thresholds to a greater extent than either P-SCS or sham. In line with preclinical models, this offers a novel HF-SCS MOA, possibly explained by the differential recruitment of larger fibers (Youn et al., 2015). In as much, QST has the potential to elucidate novel PF-SCS MOA. Dynamic QST has recently emerged as a potential method of clarifying SCS MOA as it evaluates temporal summation (TS) and conditioned pain modulation (CPM). TS, a phenomenon whereby repeated stimuli result in increased pain, is associated with central sensitization (Vierck Jr et al., 1997). Thought to gauge DAS function and correspondent to diffuse noxious inhibitory control (DNIC), CPM is a phenomenon whereby a second noxious stimulus applied elsewhere decreases pain perception from the initial pain location (Le et al., 1992; Le Bars et al., 1979). Interestingly, while Campbell et al. (2015) were unable to demonstrate a change in QST thresholds to P-SCS, they found that patients who exhibited enhanced TS and reduced CPM at baseline reported decreased pain scores after three months of P-SCS (Campbell et al., 2015). Further supporting these findings, Eisenberg et al. (2015) demonstrated that P-SCS attenuates TS and leads to improved self-reported pain (Eisenberg et al., 2006; Eisenberg et al., 2015). Together, while multiple studies have demonstrated the utility of QST and dynamic QST as objective sensory markers, there still exists wide variability in clinical data. Though this variability is likely due to small sample size, differences in patient characteristics and methodological dissimilarities, QST and dynamic QST remain promising clinical tools that require further exploration. There is a clear need for larger, more rigorous, randomized controlled trials to determine if QST metrics improve candidate selection and guide device programming for P-SCS, PF-SCS and DRG-S paradigms.

**Table 4:** Summary of QST clinical research investigating spinal cord stimulation (SCS) from inception to present

Mechanical						Thermal			Electrical				
Study Authors	Year	SCS Paradigm	Sample Size	Detection Threshold	Pain Threshold	Detection Threshold	Pain Threshold	Pain Tolerance	Detection Threshold	Pain Threshold	Pain Tolerance	Temporal Summation	Comments
Shealy et al. (1970)	1970	P	6	↔									Hyperalgesia to pinprick after P-SCS but deep pressure less painful
Larson et al. (1974)	1975	P	18	↑	↑								Changes returned to control values within 30–60 min of cessation
Lindblom et al. (1975)	1975	P	5	↑	↔								
Doerr et al. (1978)	1978	P	8	↑					↑	↑			
Marchand et al. (1991)	1991	P	8			Warm: ↑	Warm: ↑						
Mironer et al. (2000)	2000	P	44						↔	↔	↑		Tolerance correlated to success of trial stimulation and permanent P-SCS implant
Alo et al. (Aló & Chado, 2000)	2000	P	16						↑		↔		
Kemler et al. (2001)	2001	P	24		↔	Warm: ↔ Cold: ↔	Warm: ↔ Cold: ↔						Mechanical detection thresholds returned to baseline after 3 months
Cata et al. (2004)	2004	P	2	↓			Warm: ↔ Cold: ↔						↓Sharpness detection
Eisenberg et al. (2006)	2006	P	13	↑		↔	↔			↑			
Rasche et al. (2006)	2006	P	7	↓	↔	Warm: ↓ Cold: ↓	Warm: ↔ Cold: ↔						Thermal and mechanical detection and pain thresholds not statistically significant in unaffected limb
Van Eijs et al. (2010)	2010	P	24										Brush evoked allodynia is a negative prognostic factor of SCS treatment
Burkey et al. (2010)	2010	P	1			Warm: ↑ Cold: ↔							
Munster et al. (2012)	2012	P	1	↓	↔	Warm: ↑ Cold: ↓	Warm: ↑ Cold: ↓						
Meier et al. (2015)	2015	P	14	↔	↔	↔	↔						No change in wind-up
Ahmed et al. (2015)	2015	P	19			Warm: ↑	Warm: ↑	Warm: ↑					
Youn et al. (2015)	2015	HF	20	↑	↑	↔	↔						
Eisenberg et al. (2015)	2015	P	18									**↓**	↓TS at most painful site of affected leg; no effect on nonpainful leg
Campbell et al.(2015)	2015	P	24		↔		Heat: ↔	Heat: ↔				↓	↑TS and ↓CPM associated with ↓ self-reported pain
Biurrun Manresa et al.(2015)	2015	P	17							↔			↑ withdrawal reflex threshold; psychologic scores were predictors for electrical pain tolerance

Modern QST and dynamic QST has evolved considerably since the first trials demonstrating changes in threshold and tolerance. Despite early evidence demonstrating increased threshold and tolerance with P-SCS, recent studies diverge and are remarkably heterogenous in their findings. This is likely due to changes in methodology, equipment, patient factors and the evolution of SCS. Regardless, QST and dynamic QST offer a potentially exciting future avenue for investigation. Basic science applications include clarifying analgesic mechanisms of P-SCS and PF-SCS. Clinical application of QST may prove a useful tool for identifying SCS candidates and tailoring therapy to a particular patient or pain syndrome. ‘Up’ and ‘down’ arrows represent increases or decreases, respectively, in threshold or tolerance. ‘Sideways’ arrows represent no significant difference. Acronyms: P (P-SCS, paresthesia or tonic spinal cord stimulation), HF (HF-SCS, high frequency or kilohertz frequency spinal cord stimulation), TS (Temporal Summation), CPM (Conditioned Pain Modulation).

## Neurophysiologic testing

**Fig. 7:**
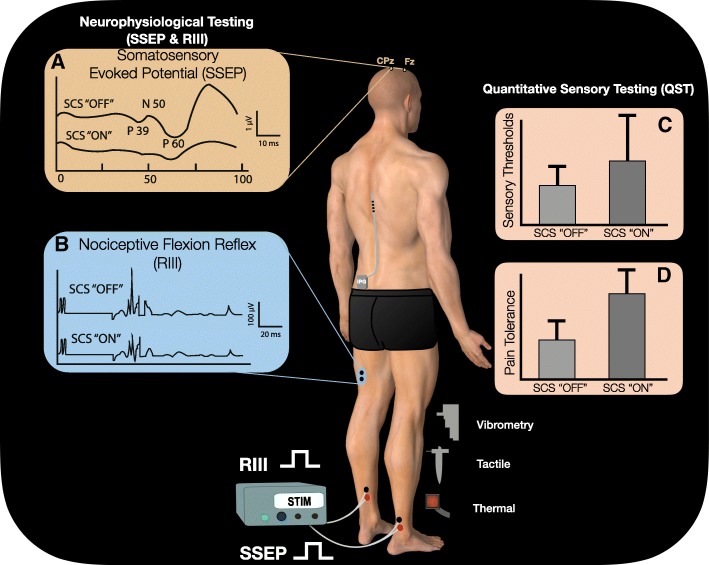
Neurophysiological testing. Somatosensory evoked potentials (SSEP) waveforms are measured by applying an electrical stimuli to the tibial nerve. SSEP waveforms recorded with scalp electrodes at (CPz-Fz) are modulated with SCS. Panel **a**: During SCS ON P39-N50-P60 representative SSEP waveform decreases in amplitude. Flexor reflexes (RIII) are obtained with noxious electrical stimuli are applied to the sural nerve. The RIII waveform is recorded from the ipsilateral biceps femoris. Panel **b**: During SCS ON there is decrease the amplitude of the RIII waveform (adapted from (Sankarasubramanian et al., 2018b)). Quantitative sensory testing (QST) applies different sensory stimuli, such as vibratory, tactile or thermal stimuli to the subject extremity. Panels **c**, **d**: SCS results in variable effects on QST sensory thresholds and may increase increases pain threshold and tolerance. Advanced QST measures including temporal summation (TS) and conditioned pain modulation (CPM) can provide further insight into P-SCS, PF-SCS and DRG-S analgesic mechanisms

The neurophysiological effects of P-SCS on the human spinal cord has been a crucial area of study in understanding the mechanistic properties of the therapy, as well as furthering its development (Sankarasubramanian et al., 2018a). The effect of P-SCS in cortical processing can be measured via ﻿somatosensory evoked potentials (SSEPs), or the activity in the cortex measured via EEG that results from peripheral electrical stimulation (Fig. 7). Using this paradigm, numerous studies have demonstrated that P-SCS can have an inhibitory effect on the amplitude of the SSEP in response to noxious stimuli, which in turn would modify the experience of the painful sensation (Wolter et al., 2013). Additional research has looked into the effect of P-SCS on the sensorimotor reflexes mediated by Aβ, Aδ, and C sensory afferents (Sankarasubramanian et al., 2018a; De Andrade et al., 2010). In a study of 20 patients, the authors demonstrated that P-SCS ﻿attenuated the H-reflex, a monosynaptic arc in the spinal cord, such as the Achilles tendon reflex, as well as the RIII, a polysynaptic withdrawal reflex, such as withdrawing one’s hand from a hot surface. Furthermore, it was shown that in this treatment group, the attenuation of the RIII correlated with pain relief from the P-SCS. This finding was also supported by other studies (García-Larrea et al., 1989; Manresa et al., 2015).

## SCS affects cortical and subcortical pain processing

### Cortical and subcortical signatures of pain

**Fig. 8:**
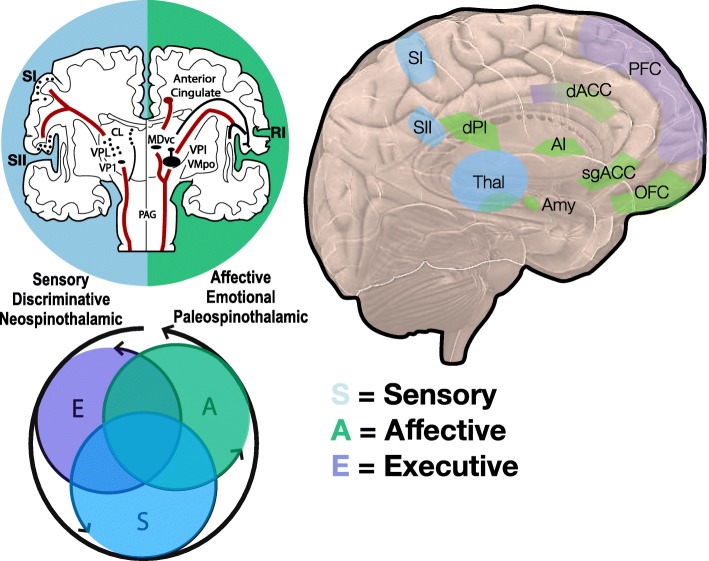
Cortical and Sub-cortical Pain Processing. Sensory-discriminative pain processing with thalamus, SI, SII and posterior IC, with sensory discriminative pain relayed through the ventroposterior-lateral and ventroposterior-medial thalamic nuclei and is also termed the neospinothalamic pathway. Affective-emotional pain processing with dorsal ACC and Anterior IC while affective sensory information to the AEN is thought to be relayed through the medio-dorsal thalamus, also termed the paleospinothalamic pathway. PFC (Prefrontal Cortex), OFC (orbitofrontal cortex) SI (Primary Somatosensory Cortex) SII (Secondary Somatosensory Cortex) dACC (Dorsal Anterior Cingulate Cortex) Amy (Amygdala), AI (Anterior Insula), RI (Rostral Insula) dPI (Dorsoposterior Insula), PAG (Periaqueductal Gray)

Despite the advent of PET, fMRI, SPECT, MEG and high-density EEG, the neural representation of nociception and the experience of acute and chronic pain remains ill-defined (Mouraux et al., 2011). Acute nociception is an alerting response. This alerting function engages salience networks that in the past were ascribed as part of the “Pain Matrix”. Recent work refute inclusion of these salience neural network nodes (that are equally engaged with alternative sensory modalities, i.e., a blaring auditory stimuli) when examining the salience of a pain percept (Mouraux et al., 2011). Nonetheless, chronic pain consistently activates areas such as the primary and secondary somatosensory cortex (SI, SII), thalamus and prefrontal cortex (PFC) as well as salience network nodes including the insular cortex (IC), dorsal Anterior Cingulate Cortex (dACC) (that may in part amplify the attention paid to chronic pain symptom, i.e., a perpetual “alerting” signal) (Apkarian et al., 2005; Barad et al., 2009). Beside the salience network, the neural representation of nociception is described as the sensory-discriminative network (SDN) and the affective-emotional network (AEN). The SDN is composed of the thalamus, SI, SII and posterior IC, with sensory discriminative pain relayed through the ventroposterior-lateral and ventroposterior-medial thalamic nuclei and is also termed the neospinothalamic pathway. The AEN is composed of the dACC (area 24), the rostral (dorsoposterior IC) and Anterior IC while affective sensory information to the AEN is thought to be relayed through the medio-dorsal thalamus, also termed the paleospinothalamic pathway (Barad et al., 2009) (Fig. 8). Prior basic science and clinical work confirm the mediodorsal thalamus is important in the processing of emotion (Metzger et al., 2010), affective pain processing (pain unpleasantness) (Metzger et al., 2010; Brooks & Tracey, 2005; Ploner et al., 1999; Rainville et al., 1997; Vogt & Paxinos, 2014; Vogt et al., 1979), thought to occur through mediodorsal thalamic connections with dorsal ACC (area 24). In a demonstrative case study, a patient with an isolated somatosensory cortex stroke that spared the dorsal ACC (area a24) and thalamus (including mediodorsal thalamus) reported usual contralateral limb analgesia to painful stimuli, but the patient continued to report an “unpleasant” feeling with the application of painful stimulus, suggesting in vivo separation of the affective and sensory discriminative pain pathways (Ploner et al., 1999). Using machine learning and fMRI to evaluate thermal pain, social pain and remifentanil response, Wager et al. demonstrated > 90% sensitivity and specificity in using neurological signatures that identified reproducible pain patterns in the thalamus, posterior and anterior IC, SI/SII, ACC and PAG (Wager et al., 2013), providing an ample construct for the involvement of the IC and ACC in the pain experience. In support of this construct, a 2016 metanalysis of fMRI and PET neuroimaging demonstrated that the anterior IC, ACC and thalamus were highly conserved in pain processing, irrelevant of imaging modality measures or the pain source (i.e., body part) (Jensen et al., 2016). Moreover, a 2005 metanalysis discerned a difference between pain experience of acute and chronic pain: acute pain stimuli in healthy control subjects were consistent with activation of the SDN while the chronic pain patients exhibited greater activation of the prefrontal cortex, thought to reflect an increase in cognitive, emotional, and introspective components (critical to AEN) (Apkarian et al., 2005; Jensen et al., 2016). Although discrete regions may perform modality specific pain processing functions, there remains wide overlap in neural activation between acute and chronic neuropathic and nociceptive pain (Barad et al., 2009). Chronic neuropathic pain, partially due to spinal pathophysiologic processes, is known to similarly result in pathological neurodegenerative process in cortical and subcortical structures. In 2004, Apkarian et al., showed that patients with chronic low back pain exhibited atrophy of the dorsolateral PFC and thalamus (Apkarian et al., 2004) demonstrative of chronic pain regional morphometric changes. Moreover, they showed that patients with neuropathic pain exhibited greater volume loss than those with non-neuropathic pain and that the duration of pain correlated with volume loss, indicating the presence of progressive degeneration over time (Apkarian et al., 2004). Further, chronic pain patients compared to controls demonstrate thalamic volumetric and morphometric changes as well as decreased activation in parts of the PFC, which may represent decreased activation of descending inhibition, or neuropathy dependent reduction in utilization due to diminution of afferent signaling (Jensen et al., 2009; Jensen et al., 2012; Segerdahl et al., 2015). Collectively pathological processes that contribute to chronic pain are pervasive throughout the neuraxis, (spinal cord and brain) that integrates sensory discriminative as well as emotional and affective pathways (Fig. 8).

### Cortical and subcortical processing with SCS

Functional imaging techniques, such as positron emission tomography (PET), functional magnetic resonance imaging (fMRI) and Magnetoencephalography (MEG) have been used to investigate neural activation and/or attenuation pre-to-post P-SCS, (Moens et al., 2013; Moens et al., 2012; Schulman et al., 2005; Oluigbo et al., 2012; Nihashi et al., 2004; Kunitake et al., 2005; Pahapill & Zhang, 2014; Kishima et al., 2010; Stančák et al., 2008; Nagamachi et al., 2006; Sufianov et al., 2014; Elaine et al., 1997; Hosobuchi, 1985; Deogaonkar et al., 2016) while relatively recent work has begun to systematically compare the neural effects of P-SCS versus PF-SCS with fMRI and PET. Moens et al. (2018) showed that there are clear differences between P-SCS and PF-SCS. First, with a randomized block design, they employed paresthesia based low frequency (4 Hz, 60 Hz) and paresthesia based HF-SCS (500 Hz and 1 kHz) compared prior to subthreshold or PF-SCS (4 Hz, 60 Hz, 500 Hz and 1 kHz). At all frequencies, PF-SCS resulted in greater cortical (frontal brain regions: limbic, sensory, and motor as well as diencephalon) activity than subthreshold (below paresthesia) PF-SCS/HF-SCS (De Groote et al., 2018). Interestingly, P-SCS resulted in greater deactivations in parahippocampus, amygdala, posterior cingulate gyrus (PCG), precuneus and superior temporal gyrus, when compared to decreases with subthreshold PF-SCS/HF-SCS (De Groote et al., 2018). Moens et al. (2018), postulated that P-SCS deactivation of parahippocampus and PCG, both with reciprocal connections to default mode network (DMN) targets that include the dorsal thalamus and inferior parietal lobe (Buckner et al., 2008), could be clinically relevant, given that dysfunctional DMN connectivity occurs in chronic lower back pain (Baliki et al., 2008; Letzen & Robinson, 2017), complex regional pain syndrome (Bolwerk et al., 2013), and failed back surgery syndrome (Letzen & Robinson, 2017; Kornelsen et al., 2013). In line with these results, Deogonaker et al. (2016) reported increased DMN connectivity with optimal P-SCS, further supporting that P-SCS may correct dysfunctional DMN connectivity associated with chronic pain (Deogaonkar et al., 2016). Most recently, Moens et al., (2019) re-examined (fMRI) network functional connectivity (FC) during the resting state pre-to-post implant with paresthesia free HF-SCS (at 10KHz) at one and three months (De Groote et al., 2019). Interestingly, HF-SCS patients demonstrated an increase in FC between the right anterior insula (RAI) and both left lateral and dorsolateral PFC (LPFC and DLPFC). The post HF-SCS increase in FC of RAI to PFC is posited to indicate increases in central executive network (CEN) activity known to be dysregulated (decreases in FC and CEN activity) in chronic affective pain (Jiang et al., 2016) and inversely correlated to increased pain catastrophizing reports (Jiang et al., 2016). Collectively, the emerging evidence shows P-SCS and PF-SCS may differentially correct dysfunctional neural networks ubiquitous in the chronic pain patient.

**Fig. 9:**
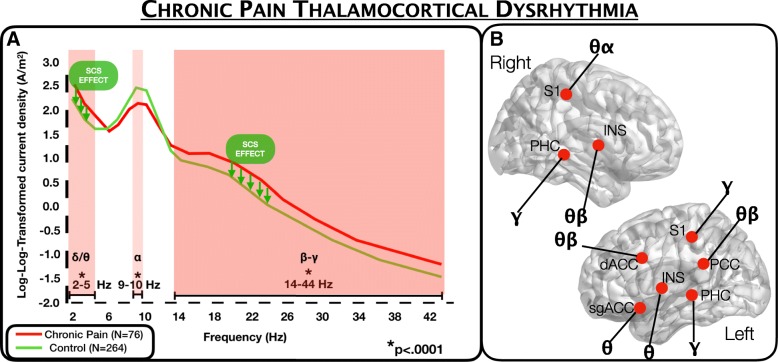
Chronic pain thalamocortical dysrhythmia is known to occur in distinct band, i.e. most commonly in theta, beta, and gamma bands. Work from De Ridder and Vannesste demonstrate *p* < .0001) with greatest differences found in theta-beta theta and gamma (Panel **a**), source localized to bilateral sensory discriminative pathways SI, which additional affective emotional pathways including dACC, sgACC, bilateral INS, bilateral PHC, and posterior cingulate cortex (Panel **b**). Pretrial and or implant characterization of chronic pain patient TCD could inform the practitioner of an optimal SCS paradigm, while post SCS TCD measures could track SCS efficacy (i.e., decrease in predominate θ theta, β beta, γ gamma dysrhythmias) as described by Schulman et al. (2005). dACC dorsal anterior cingulate cortex, sgACC subgenual anterior cingulate cortex, INS insula, PHC parahippocampus, SI primary somatosensory cortex, PCC posterior cingulate cortex, θ theta, α alpha, β beta, γ gamma. Figure adapted from Vanneste et al. (2018) Nature Communications (Vanneste et al., 2018)

Beside functional magnetic resonance imaging and volumetric measures, thalamocortical dysrhythmia (TCD) has been proposed as an underlying mechanism of chronic neuropathic pain and other pain disorders (Jensen et al., 2013; Llinas et al., 2005; Llinas et al., 1999; Sarnthein & Jeanmonod, 2008; Stern et al., 2006; Vuckovic et al., 2014; Walton et al., 2010). TCD is due to inhibitory asymmetries resulting from activation of cortical inhibitory interneurons at variable frequencies (Llinas et al., 2005; Walton et al., 2010). TCD measured by MEG (Rainville et al., 1997; Vogt & Paxinos, 2014; Vogt et al., 1979) and EEG (Vanneste et al., 2018), predominantly shows enhanced low frequency theta (5–8 Hz), as well as higher frequency beta (13–30 Hz) and gamma (30-60 Hz) power when chronic neuropathic pain patients were compared to healthy control subjects (Fig. 9). It is important to point out that high frequency thalamocortical oscillations underlie conscious states (beta 13–30 Hz, and gamma 30–60 Hz), whereas increased power in persistent low-frequency (theta 5–8 Hz and delta 1–4 Hz) activity, does not. Polymorphic low-frequency rhythms can result from brain lesions that interrupt important afferent inputs to the gray-matter of cortex, either by white matter, thalamic, hypothalamic or brainstem lesions, that suggest cortical slow wave activity results from cortical deafferentation (Ball et al., 1977; Gloor et al., 1977). Abnormal low-frequency rhythms can also be induced by the administration of atropine (Schaul et al., 1978). Atropine is a competitive antagonist of acetylcholine receptors and can block or limit the action of ACh. Together these animal experiments concluded that cortical deafferentation was a key factor in abnormal low-frequency activity, owing to inhibition of the cholinergic pathway (Schaul, 1998). Interestingly, persistent low frequency thalamocortical oscillations initially thought to only be present during dreamless sleep (N3 stage) and or due to cortical deafferentation, have now also been observed in neurological and psychiatric conditions (chronic pain and schizophrenia) during wakefulness and in the absence of a structural lesion (Llinás et al., 2005). Moreover, patients with neuropathic pain demonstrate TCD phase amplitude coupling and coherence between low frequency theta and higher frequency beta bands localized to cortical pain processing centers, including the PFC, ACC and sgACC, insular cortices as well as primary (SI) and secondary somatosensory (SII) cortices (Stern et al., 2006). Functional neurosurgical lesioning of the thalamic central lateral nucleus leads to reversal of overactive TCD coherence (theta gamma and theta beta) and reduction in reported pain that further supports TCD as central to pain chronicity (Stern et al., 2006). Additional work by Shulman et al. (2005) has shown that theta power significantly decreased to level of healthy controls in successfully P-SCS treated CRPS patients while unsuccessful P-SCS showed similar theta power to untreated deafferentation pain syndrome patients (Schulman et al., 2005). Expanding on this work, Vannesste and De Ridder et al. (2018) studied source localized TCD in 78 chronic pain patients (Vanneste et al., 2018) and showed: 1) increases in insular cortex and sgACC theta band power, 2) increases in SI and parahippocampus gamma band power, and 3) increases in theta/beta coherence in dorsal anterior, posterior cingulate and insular cortex (Vanneste et al., 2018) (Fig. 9). In a small cohort, this group found that B-SCS decreases: 1) theta and gamma band power in bilateral SI, 2) alpha and beta power in dorsal anterior, posterior cingulate, 3) theta power in the pgACC, and 4) in phase coherence between dACC and SI as well as between sgACC and SI (Vanneste & De Ridder, 2018). Taking into account that chronic pain patients exhibit TCD (Theta, Beta and Gamma band increases in power and coherence) there is a growing consensus that TCD could be used as a biomarker of the effects of P-SCS vs. PF-SCS while pre-treatment coherence and power could be predictive of SCS efficacy (De Ridder et al., 2013; Koyama et al., 2018). Emerging preclinical work from Saab et al. (2018), demonstrates that PF-SCS produced a reduction of theta band power in a CCL neuropathic pain model. The authors argue that reduction in theta band power could be used as a marker for PF-SCS efficacy. De Ridder et al. (2014) demonstrated that B-SCS resulted in activation of dorsal ACC with increases in beta and alpha power (De Ridder et al., 2013) and postulated B-SCS may preferentially modulate the paleospinothalamic pathway. Yearwood et al. (2016, 2019) also demonstrated that B-SCS modulates the posterior anterior cingulate and subgenual cortex (Yearwood et al., 2019; Yearwood et al., 2016). Intriguingly, Quindlen-Hotek et al. (2019) demonstrated that B-SCS increased dorsal anterior cingulate firing frequency in a in a rat nerve root compression model (Quindlen-Hotek et al., 2019). In a recent small cross-over study, our group, showed that a 5 day treatment with active charge balanced B-SCS resulted in: 1) reduction gamma and beta band power in the mediodorsal thalamus, and 2) a significant reduction in theta band power within SI SII and bilateral dorsal, mid and anterior insular cortices (Lerman et al., 2019), further supporting B-SCS unique cortical and subcortical effects on TCD in chronic pain patients (De Ridder et al., 2013; Yearwood et al., 2016; Quindlen-Hotek et al., 2019). Although no clinical studies have been completed, preclinical work by Pawela and Hogan et al. (2017) have shown DRG-S attenuates fMRI BOLD neospinothalamic and paleospinothalamic response, including SI, SII, retrosplenial granular cortex, thalamus, caudate, putamen, nucleus accumbens, globus pallidus, and amygdala (Tang et al., 2014). Together, variations in pain processing patterns with functional neuroimaging suggest central maladaptive neuroplasticity in chronic pain patients contributes to the chronic pain experience while rich areas of research that investigate the neural effects of neuromodulation therapies (including P-SCS, PF-SCS and DRG-S and emerging ECAP-SCS) remain to be explored (Jensen et al., 2016).

## P-SCS and PF-SCS mechanisms: evidence and theory

### Paresthesia-based spinal cord stimulation (P-SCS)

Premised on the gate-control theory of pain, the original hypothesized mechanism of P-SCS was that stimulation of Aβ fibers and inhibitory interneurons led to inhibition of pathologic WDR neuron firing to Aδ and C fiber inputs (Melzack & Wall, 1965; Sivanesan et al., 2018). It is now understood that multiple mechanisms contribute to P-SCS analgesia. Applied with a “low” frequency between 40 and 60 Hz and PW of 150–500 μs, amplitude and lead position are adjusted until the patient feels a tolerable, non-painful paresthesia covering the target dermatome. Indicated for neuropathic pain syndromes (Linderoth & Foreman, 2017), P-SCS is the most extensively studied paradigm and boasts the largest body of evidence. Preclinical and clinical evidence for SCS efficacy is largely based on P-SCS and thus its mechanisms will be summarized here. At the spinal segmental level, a delivered pulse antidromically activates dorsal horn fibers as well as generates an electric field capable of axon depolarization in the vicinity of the dorsal cord (Yakhnitsa et al., 1999; Tazawa et al., 2015). It is postulated that antidromic activation of Aβ fibers contributes to retrograde activation of inhibitory interneurons which exert presynaptic control over PN firing (Zhang et al., 2014). While initially DH mapping, dorsal root potentials and TENS efficacy seemed to confirm this mechanism, recently the simplicity of these DH neural networks has come into question (Mendell, 2014; Szentagothai, 1964; Hochman et al., 2010). The activation of DH inhibitory interneurons clearly plays a role in P-SCS-induced antinociception, as the lamina II inhibitory plexus arborizing to PN is the major source of GABA and glycine in the DH (Keller et al., 2001; Yasaka et al., 2007). With P-SCS, an increase in DH GABA and decrease in glutamate via presynaptic GABA inhibition is critical to improvement in allodynic rat models (Yakhnitsa et al., 1999; Cui et al., 1996; Stiller et al., 1995) (Fig. 4). However, the origin of inhibitory interneuron activation remains uncertain. Currently, it is unclear the degree to which descending inputs, antidromic activation via Aβ afferents and direct modulation by a localized electric field each affect the inhibitory action of interneurons (Elbasiouny & Mushahwar, 2007; Francis et al., 2003; Jefferys et al., 2003). Studies of intact, decerebrate and cord-transected models each demonstrate efficacy with SCS, arguing for multiple contributions to the dorsal horn inhibitory circuit (Saade et al., 1985; Saade et al., 1986).

P-SCS may attenuate gliosis and glial-mediator release, inflammatory and maladaptive responses to neural injury contributing to allodynia and hyperalgesia (Ji et al., 2013). Closely interlinked with neurons in the proposed tripartite synapse, glia are responsible for contributing input via secretion of bioactive mediators which results in increased excitatory and decreased inhibitory post-synaptic currents after nerve injury (Fig. 6) (Mika et al., 2013; Araque et al., 1999). Modulation of gliosis, glial-mediator release and neuroinflammation is a largely unexplored potential mechanism of P-SCS-mediated analgesia. Currently, the limited body of evidence suggests low frequency P-SCS decreases microglia and astrocyte activation, though little is known about changes in local glial mediators (Sato et al., 2014; McCarthy & McCrory, 2014a). Some work has been performed evaluating peripheral inflammation in CRPS patients showing reduced cytokine levels measured in the peripheral tissues, but it is unclear whether this work will translate to the DH (Kriek et al., 2018). Given that P-SCS likely reduces gliosis and peripheral inflammation, SCS attenuation of gliosis and glial mediator release is an exciting area for further investigation.

Orthodromic activation of the dorsal columns also facilitates antinociception through triggering of supraspinal inhibitory loops, colloquially referred to as the serotonergic and noradrenergic DAS (Fig. 5) (Saade et al., 1986; Tazawa et al., 2015). Stimulation of these descending pathways leads to increases in DH serotonin and NE, which limit PN firing through a GABAergic mechanism. Activation of the serotonergic DAS by P-SCS leads to increased DH 5-HT, and subsequently GABA and glycine (Cui et al., 1997; Stiller et al., 1996; Cui et al., 1996). It is likely that the serotonergic DAS plays a greater role in P-SCS-mediated antinociception than the noradrenergic DAS, the role of which needs to be further clarified (Song et al., 2013b). Additionally, the analgesic effect of opioids likely includes the recruitment of the noradrenergic DAS (Tazawa et al., 2015). Opioidergic mechanisms contribute to P-SCS-mediated analgesia with frequency-dependent, differential activation of opioid receptor subclasses (Sato et al., 2013). Moreover, there may be a development of tolerance to opioidergic recruitment as well as an opioidergic ceiling effect of maximal benefit, which should be further investigated (Chandran & Sluka, 2003; Inoue et al., 2017). The site of action and mechanism of P-SCS opioidergic recruitment remains to be determined. While the presence of the DAS and supraspinal relationships continue to be heavily investigated, the DH neural circuitry, and relative contribution of descending targets remain to be fully elucidated. Though our understanding of the involvement of segmental and supraspinal mechanisms of P-SCS-induced analgesia has dramatically improved, further research is needed to clarify the role and necessity of each of these mechanisms in regard to overall antinociception.

### High frequency spinal cord stimulation (HF-SCS)

Paresthesia-free high frequency SCS (HF-SCS) refers to kilohertz-frequency impulses delivered via percutaneously placed epidural electrodes. Having level I evidence for patients with back or leg pain, HF-SCS at 10 kHz showed superiority to P-SCS in two separate randomized clinical trials (Kapural et al., 2015; Kapural et al., 2016). In stark contrast to P-SCS, HF-SCS employs a paresthesia-free charge delivery strategy exploiting the strength-duration curve, delivering high frequency and low amplitude pulses to maximize total charge delivery without generating a paresthesia (Kapural et al., 2015). Pain relief with HF-SCS does not correlate to territory of paresthesia, supporting a novel MOA. Further evidence for this stems from clinical data demonstrating early pain relief with traditional SCS compared to delayed relief with kHz frequency stimulation (Chakravarthy et al., 2018b). While 10 kHz stimulation is the most commonly employed delivery strategy, evidence for efficacy with sub-threshold lower-frequency paradigms including 1 kHz, 1.15 kHz, and 5 kHz have demonstrated benefit over P-SCS (Youn et al., 2015; North et al., 2016; Perruchoud et al., 2013). In 2018, the PROCO RCT published their data on HF-SCS, finding no difference in outcomes for interval frequencies from 1 to 10 kHz (Thomson et al., 2018). Though the mechanisms of pain relief with HF-SCS remain to be elucidated, Linderoth et al. (2017) evaluated 3 working hypotheses initially presented at the 2016 Neuromodulation: The Science meeting (Linderoth & Foreman, 2017). These listed hypothesis report HF-SCS results in: 1) a reversible depolarization blockade, 2) desynchronization of neural signals and 3) membrane integration, although others propose a mechanism of glial-neuronal modulation (Linderoth & Foreman, 2017; Chakravarthy et al., 2018b). While depolarization blockade and membrane integration offer a reasonable physiologic explanation of SCS relief, Lempka et al. (2015) employed neuronal modeling that demonstrated direct activation or conduction block of DH or DRG neurons required a higher amplitude than the current clinical devices are capable of delivering (Lempka et al., 2015). In support of this assertion, follow up preclinical work demonstrated a lack of DH block *as well as a* lack of DH activation with sub-threshold HF-SCS (Song et al., 2014). Crosby et al. (2016) showed that only supra-motor threshold HF-SCS resulted in a reliable antidromic or orthodromic AP recording, further suggesting HF-SCS must employ alternative mechanism to known Hodgkin-Huxley neuronal models (Crosby et al., 2016). Similarly, Song et al. (2014) showed sub-threshold HF-SCS is unable to evoke action potentials to DH nuclei and instead proposed that its MOA is likely segmental and does not involve the supraspinal mechanisms seen in P-SCS (Song et al., 2014). Consistently demonstrated in both preclinical work and clinical experience, the antinociceptive effects of HF-SCS inevitably have a delayed onset in comparison to P-SCS (Shechter et al., 2013). Having taken this delay into account, McMahon et al. (2016) applied 20% motor threshold at 10 kHz frequency in close proximity to DC that showed significant reduction in windup of superficial DH PN (McMahon & Smith, 2016). Of note, applying 20% motor threshold to the DC does not activate Aβ-fibers and is also very unlikely to generate any relevant paresthesia in clinical settings. Using the same SCS paradigm, Kagan et al. (2018) showed treatment with HF-SCS at 20% motor threshold reduced DH PN activity with depressed firing up to 4 min after HF-SCS had ceased (Kagan et al., 2018). Further, Li et al. (2018) showed that application of 500 Hz DC stimulation increased DH c-Fos, providing further support that HF-SCS modulates dorsal horn neural circuits (Shiyeng et al., 2018). While the effects of HF-SCS on DH circuitry have been somewhat clarified, the mechanisms by which these changes occur remain nebulous. Of particular note, work by Zannou et.al. (2019) demonstrates that HF-SCS applied at 10 kHz resulted in significant local heating (Zannou et al., 2019). The authors postulate that local temperature increase in response to HF-SCS may result in thermal homeostatic changes and potentially provides an explanation for the delayed onset of pain relief by HF-SCS (Thomson et al., 2018; Al-Kaisy et al., 2015). Specifically, they hypothesize HF-SCS modulates neuroinflammation through changes in 72 kDa heat shock protein (Hsp70), known to inhibit activation of proinflammatory transcription factors in SGC (Zannou et al., 2019). Together, converging lines of evidence support the direct effect of HF-SCS on local dorsal structures in the spinal cord, likely contributing to the clinical effects of HF-SCS. While the effects of P-SCS on glial-neural modulation have been demonstrated, the effects of HF-SCS on glial synaptic modulation remain to be studied. Moreover, to date there is a relative absence of literature evaluating the supraspinal mechanisms of HF-SCS, including those measured by neuroimaging or direct measurement of neurotransmitters and local mediators. Further work is clearly needed to elucidate these mechanisms.

### Burst spinal cord stimulation (B-SCS)

**Fig. 10:**
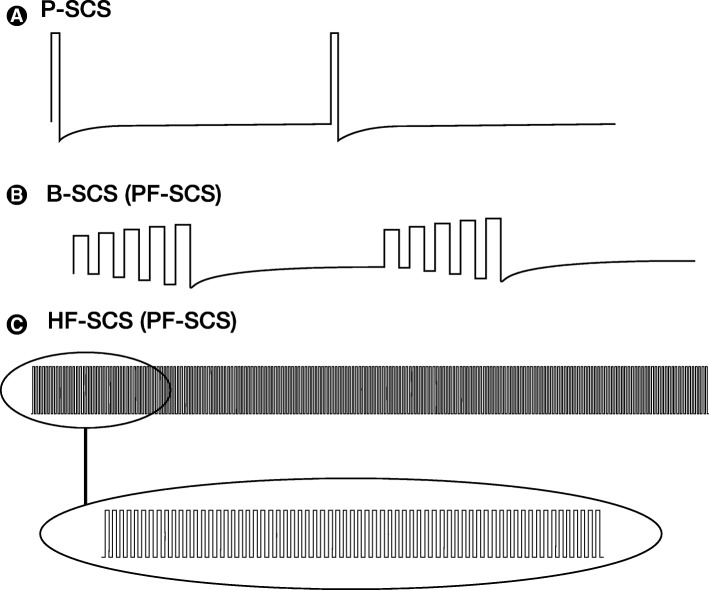
Stimulation Patterns in Paresthesia and Paresthesia Free SCS Paradigms: Traditional SCS comprised of tonic or repetitive low frequency SCS usually in the range of 40–60 Hz produces paresthesia of P-SCS (**a**). Paresthesia free, burst spinal cord stimulation (B-SCS) employs incremental increase in amplitude with each burst. Inter-burst frequency is 40 Hz, while intra-burst frequency is 500 Hz (**b**). Between each burst there is a passive recharge phase. Paresthesia free high frequency SCS employs an ultra-high frequency of 10 kHz in continuous mode (**c**)

Predominantly paresthesia-free in the majority of patients with neuropathy, B-SCS employs a stacked pulse paradigm for charge delivery (De Ridder et al., 2010; Deer et al., 2018; Schu et al., 2014; Tang et al., 2014) (Fig. 10). Endogenous neuronal burst firing is ubiquitous throughout the neuraxis and results in activation of either subthreshold membrane conductance initiating AP or a supra-threshold membrane conductance that once activated evokes two or more AP. These high-frequency AP occur during a plateau or active phase and are followed by a period of relative quiescence, termed the silent phase. During the silent phase, slow Ca^2+^ channel opening results in threshold depolarization via Na^+^ and Ca^2+^-activated conductance that is inherent to neuronal burst firing. Relevant neuronal targets that exhibit burst firing have consistently been identified in the mediodorsal thalamic (Hodaie et al., 2006) and dorsal horn neurons (Russo & Hounsgaard, 1996; Lopez-Garcia & King, 1994), suggesting that neuronal burst firing plays a role in both the neo and paleospinothalamic pathways, while afferent C tactile fibers also exhibit bursting firing (Liljencrantz & Olausson, 2014). In the DH, a majority of bursting cells are found in lamina I, while bursting neurons of the dorsomedian nucleus project third order neurons to the ACC (Hodaie et al., 2006), previously shown to be modulated by B-SCS (De Ridder et al., 2013). Multiple thalamo-cortical relays demonstrate thalamic burst firing. An initial burst can result in greater ability to elicit cortical AP, while repeated burst firing raises the probability of an aggregate increase in thalamo-cortical summative signals, potentially increasing the salience of that signal (Swadlow & Gusev, 2001; Sherman, 2001). Moreover this summative signal undergoes “multiplexing”, meaning that certain neuron ensembles will preferentially activate when receiving particular burst frequencies, while other neuronal ensembles may remain quiescent (Izhikevich et al., 2003). In clinical B-SCS, frequencies consist of 40-Hz bursts with five spikes at 500-Hz spike frequency, a pulse width of 1000 μs, and an inter-burst interval of 1000 μs (Fig. 10). By design, B-SCS emulates endogenous neuronal bursting patterns. B-SCS waveforms replicate endogenous burst-firing Na^+^ spikes that ride on a Ca^2+^-dependent plateau. This eventually becomes charge balanced after the high-frequency spikes are terminated. For instance, neurons of the dorsomedian nucleus demonstrate a mean burst duration of 1000–1600 μs with approximately 3 to 5 spikes per burst that is in line with the De Ridder B-SCS paradigm (De Ridder et al., 2013). Interestingly, Crosby et al. (2015) showed larger pulse widths incrementally increase B-SCS analgesia in preclinical rat SNL models (Crosby et al., 2015b; De Ridder et al., 2018). Of note, inter-burst frequency employs the most common P-SCS treatment frequencies (i.e., 40 Hz) while the intra-burst frequency of 500 Hz parallels that of HF-SCS paradigms. In a rat SNL model treated with B-SCS, Crosby et al. (2015) determined that changes in inter-burst frequency (comparing 20, 40 and 60 Hz) did not incrementally decrease DH excitability. Interestingly, they did note that increasing the number of pulses per burst correlated with reduced DH activity (Crosby et al., 2015b). This is in line with preclinical literature that shows that increasing the number of pulses per burst: 1) incrementally increases the nonlinear buildup of the postsynaptic potential, 2) improves signal to noise ratio, and 3) results in enhanced neuroplasticity. However, less effects are seen at greater than 6–7 spikes per burst, indicating a ceiling effect (Snider et al., 1998). In support of this construct, Kent et al. (2017) further showed incremental increases in each pulse amplitude over the course of each burst resulted in summative increases in ECAP (Kent et al., 2017). Further in line with this finding, Gong et al. (2016) indicated that incremental increases in the intraburst frequency were more efficacious than P-SCS, measured with increased PWT in a rat SNL model (Gong et al., 2016). De Ridder et al. (2018) suggest that intraburst frequency of 500 Hz is critical for efficacy, citing work by Song et al. (2008) that showed higher frequency was inductive of maximal SCS opioidergic effects, although this finding has not been replicated in pre-clinical B-SCS models (Song & Marvizón, 2003). Crosby et al. (2015) further reported that increases in B-SCS amplitude incrementally decrease DH excitability. In aggregate, pulses per burst and pulse width parameters constitute the charge density, which has been shown as a predictor of B-SCS efficacy (Crosby et al., 2015b). Besides effects in the DH, Tang et al. (2014) reported P-SCS action on WDR and low-threshold (LT) neurons within the gracile nucleus; however, B-SCS had no significant impact on gracile neuronal firing (Tang et al., 2014). Nonetheless, the authors showed that B-SCS outperformed P-SCS, showing greater analgesic effects when measured with the visceromotor reflex challenge (Tang et al., 2014). Meuwissen et al. (2018) recently compared analgesic effects of B-SCS to P-SCS in a SNL rat model, while also comparing charge per second for each SCS paradigm. Introducing temporal charge domain measures, their group showed that although B-SCS was equally effective to P-SCS at lower motor threshold, B-SCS employed a relatively greater charge per second to achieve equivalent analgesic effects (Meuwissen et al., 2018). Interestingly, while segmental GABAergic effects are paramount to P-SCS efficacy, they are differentially regulated in B-SCS (Tables 1 and 2). Crosby et al. (2015) showed that although B-SCS and P-SCS both attenuated evoked WDR neuron activity to noxious stimuli, administration of a GABA_B_ receptor antagonist abolished attenuation of WDR activity in P-SCS but not B-SCS (Crosby et al., 2015b). Moreover while P-SCS increased serum GABA concentrations, this was not observed with B-SCS, indicating a diverging mechanism (Crosby et al., 2015b). While the authors accept that serum GABA levels are not necessarily reflective of CNS GABA concentrations, these results nonetheless suggest that B-SCS analgesia is likely mediated through non-GABAergic mechanisms. To date, no published studies have determined the effect of B-SCS on 5-HT, NE or endogenous opioid pathways. Though Kinfe et al. (2018) did show an increase serum IL-10 with B-SCS, it is difficult to substantiate these findings as representative of actual inflammatory-mediated change in the CNS (Kinfe et al., 2017). As mentioned, electrical pulses applied to thoracic spinal cord are not likely to directly modulate systemic effects of cytokines levels, however indirect attenuation of pain, changes in mood, or improvement in sleep may result in these observed anti-inflammatory effects (Lerman et al., 2016; Irwin et al., 2006; Irwin & Cole, 2011; Slavich & Irwin, 2014). Although it is difficult to determine the direct neuronal effects of B-SCS on supraspinal and cortical attentional mechanisms, as Tang et al., (2014) reported an absence of gracile neuronal activity, B-SCS may *modulate* dorsal column and dorsal horn activity. De Ridder et al. (2018), postulated that B-SCS 1) modulates low-threshold, *tactile* C-fibers that are known to be antinociceptive and 2) mediates multiplexing which could contribute to supraspinal analgesic and modulation of cortical attentional mechanisms (Liljencrantz & Olausson, 2014; Izhikevich et al., 2003; De Ridder et al., 2018). In support of B-SCS supraspinal cortical effects, Meuwissen et al. (2019) developed and validated the first preclinical operant motivational testing method that assessed affective-motivational aspects of pain in neuropathic rat models treated with SCS. In this model, the animal must brave a nociceptive challenge: crossing over noxious probes from an aversive brightly lit chamber to receive the reward, a dimly lit chamber. Implicating that B-SCS specifically modulates supraspinal cognitive affective-emotional circuits, they showed biphasic B-SCS improved exit time from the aversive chamber more than P-SCS, with no difference in measured PWT. This seminal work represents a truly novel preclinical model that may further translate our clinical observations. Due to remaining large knowledge gaps regarding these mechanisms, there is a clear need for the pain community to continue investigating these promising avenues (De Ridder et al., 2013; Yearwood et al., 2016; Quindlen-Hotek et al., 2019; Lerman et al., 2019). The clinical and scientific community should remain committed to clarifying the process by which B-SCS imparts clinical efficacy, with particular regard to segmental, supraspinal and inflammatory mechanisms.

### Dorsal root ganglion stimulation

Dorsal root ganglion stimulation (DRG-S) is an emerging neuromodulation target made possible by continued neurotechnology advancements, with increasingly more steerable and flexible leads (Liem et al., 2013). The intentional neuromodulation of the DRG has been described as early as 1998 and 1999 (Alo et al., 1999; Wright & Colliton, 1998). However, reports in the literature have been appearing more consistently only within the past 5 years (Harrison et al., 2018) (Hunter et al., 2019)). The DRG is located in the lateral epidural space and contains the cell bodies of the sensory neurons, crucial for the transduction of pain (Koopmeiners et al., 2013; Van Buyten, 2018) (Fig. 1). As described above, pathologic changes occur within the sensory neurons in chronic pain states, such as increased firing rates, making them a potential target for stimulation. While the evidence has shown traditional SCS can provide substantial relief for multiple painful conditions, it has limitations inherent to its physical location within the spine and its delivery of the therapy (Liem et al., 2013; Harrison et al., 2018; Deer et al., 2013). SCS electrodes are traditionally placed in the posterior epidural space. Even when considering the multiple modalities as reviewed above - traditional, high frequency, and burst stimulation – they all deliver the energy targeting the posterior spinal cord tracts, and not the sensory neurons themselves. This has been useful for coverage of an entire limb pain, and diffuse neuropathies; however, it can make coverage of specific targets difficult, such as pelvic pain, mononeuropathies, back pain, and unilateral and/or distal limb pain (Harrison et al., 2018; Van Buyten, 2018). DRG stimulation allows for more specific delivery of therapy to the affected dermatomes or pain regions.

A recent in vitro animal study of DRG-SCS demonstrated an alteration in Ca2+ influx slowed nerve conduction velocity, reduced action potential propagation and neuronal excitability as possible mechanism of action (Koopmeiners et al., 2013). In this way, DRG-S provides analgesia by blocking APs induced from the periphery, as well as the pathologic ectopic activity in the neuronal cell body. Further, Pawela et al. (2017) demonstrated changes on functional magnetic resonance imaging (fMRI) in a rat animal model, with attenuation in the regions of the brain associated with response to noxious stimuli (Koopmeiners et al., 2013; Pawela et al., 2017; Kramer et al., 2018). As compared to the control group, the response to noxious stimuli in the primary/secondary somatosensory cortex, retrosplenial granular cortex, thalamus, caudate putamen, nucleus accumbens, globus pallidus, and amygdala was attenuated with DRG-S. Interestingly, they further confirmed their findings with high-intensity (above treatment level) DRG-S, which produced a signal map similar to an acute noxious stimulation. Pan et al. (2016) studied DRG-S in an rat animal model of induced neuropathic pain from a sciatic nerve injury (Pan et al., 2016). They demonstrated that DRG-S reversed mechanical and cold hypersensitivity in the neuropathic pain state by animal behavioral response to the stimuli. Animals receiving DRG-S lacked the elevated expression of injury markers present in the positive injury control (Pan et al., 2016). Markers of glial cell activation and neuronal injury, respectively, GFAP and ATF-3 were similar between normal uninjured DRG and DRG with stimulation. This finding is remarkable, as GFAP and ATF-3 were both elevated in the positive injury control (Pan et al., 2016). Currently, there are multiple published studies that demonstrating the efficacy of DRG-S in humans. Fourteen studies were reviewed in 2018 by Harrison and colleagues, which demonstrated promising outcomes (Harrison et al., 2018). The ACCURATE RCT examined the efficacy of DRG-S compared to traditional SCS in patients with CRPS, and found DRG-S to be significantly superior in treating pain. The placement of the DRG electrodes within the neural foramen is widely accepted as being technically difficult, with a higher learning curve than traditional SCS and potentially more painful intra-procedurally (Van Buyten, 2018; Deer et al., 2013). The use of DRG-S is currently limited to practitioners who have completed specific training and demonstrated competency with the device, while its adoption is rapidly increasing in the United States.

### Closed-loop evoked compound action potentials

**Fig. 11:**
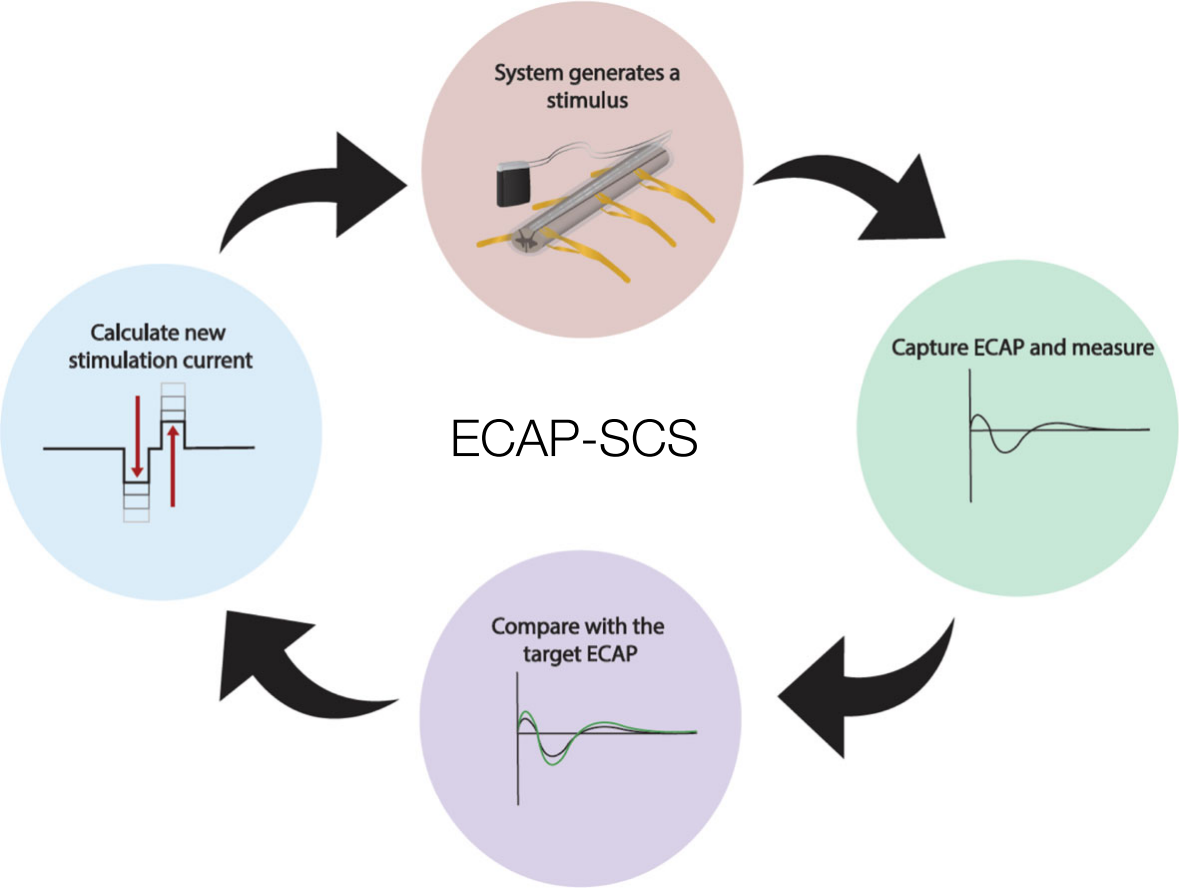
ECAP-SCS. Newly developed closed-loop SCS system measures evoked compound action potentials (ECAPs) from the spinal cord after each pulse. Greater ECAP amplitude represents more action potentials and is equivalent to increased fiber activation. Variable lead to cord contact occurs with change in position and over time. ECAP-SCS captures and calibrates SCS lead current to target desired ECAP waveform therefore minimizing variability in stimulation, which may improve clinical outcomes. ECAP-SCS is an objective measure of spinal cord activation that may help to predict responders to P-SCS

By combining the understanding of antidromic stimulation from SCS affecting SSEP, involuntary sensorimotor reflexes and correlation with treatment efficacy, further research has been done involving ECAP as a method of both studying and modifying the delivery of  SCS therapy. ECAP is the neurophysiologic recording of the response of nerve fibers to a stimulus (Russo et al., 2018; Guan et al., 2018)). More specifically, it is an ion measurement along the membranes of the nerve’s axon, which occurs during an AP, which subsequently generates the electric field to be recorded (Russo et al., 2018; Guan et al., 2018; Laird & Parker, 2013). Thus, the conduction velocity, amplitude, fiber diameter, and number of recruited fibers can be calculated (Guan et al., 2018). Additionally, the excitability and frequency of the AP can be used to infer the type of pain the patient is experiencing. For example, repetitive neuronal discharges and increases in sensory fiber excitability have been linked to chronic neuropathic conditions (Devor, 2009). The leads of P-SCS are in an ideal position to measure the ECAP of the dorsal columns as they lay just posterior in the epidural space. In this way, the non-stimulating electrodes can be modified to measure the ECAP of the axons being stimulated (Guan et al., 2018; Laird & Parker, 2013). This opens up the possibility of both studying the effects on neuronal tracts to further understand mechanisms of action of the multiple SCS paradigms, as well optimizing the treatment for the patient to improve efficacy. The feasibility of using P-SCS leads to measure ECAP was demonstrated in both an animal and human model by Parker and colleagues (Parker et al., 2013; Parker et al., 2012). Both studies confirmed the previously held theory that P-SCS primarily recruits large diameter Aβ fibers and that the ECAP amplitude increases with increasing current delivered by the leads. By utilizing this closed-loop measurement, ECAP can function as a feedback control to adjust dorsal column fiber recruitment, allowing the device to adjust delivered current in order for patients remain in the therapeutic window throughout treatment, while avoiding unnecessary and potentially uncomfortable overstimulation. While one open-label study found benefit, further study of efficacy and feasibility of this technology as a therapeutic option is needed (Russo et al., 2018) (Fig. 11).

## Conclusion

**Fig. 12:**
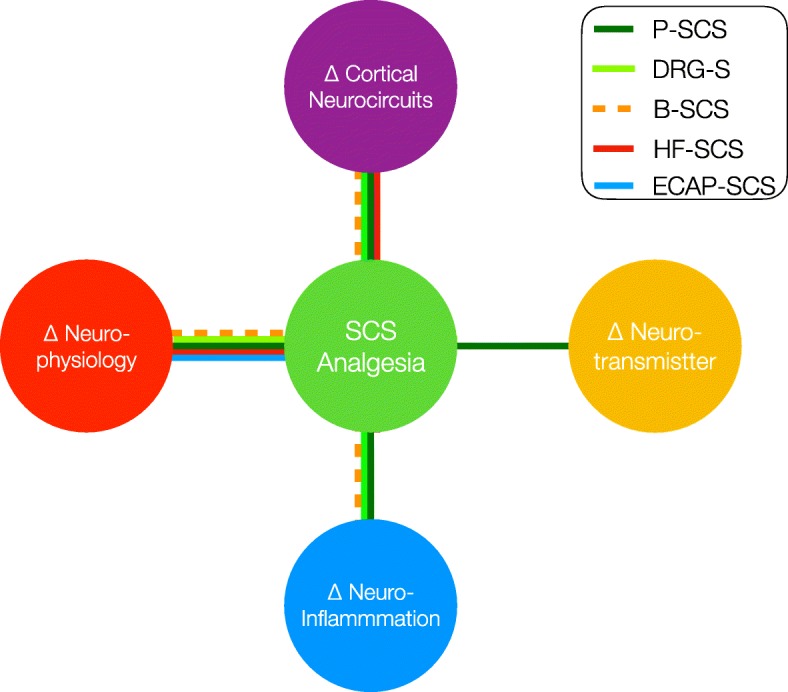
The Current literature supports multiple domains of MOA of SCS. Major P-SCS, PF-SCS and DRG-S analgesic MOA include modulation of 1) segmental and supraspinal neurotransmitters, 2) segmental and supraspinal neurophysiology/neuroplasticity, 3) central and peripheral neuroinflammation and 4) cortical and subcortical neurocircuits. P-SCS has accrued the largest literature in support of all four MOA, while only P-SCS preclinical studies consistently demonstrate modulation of neurotransmitters critical to analgesia. Emerging and published literature support the concept that all SCS paradigms (P-SCS, B-SCS, DRG-S, HF-SCS, ECAP-SCS) contribute to altered neuronal activity (i.e. neurophysiological). Pre-clinical and clinical work support cortical SCS paradigms (P-SCS, B-SCS, DRG-S, HF-SCS, ECAP-SCS) contribute to altered neurocircuit activity

With accelerated interest in novel paradigms and neural targets, the rapid evolution of SCS has helped define the field of bioelectronic medicine. This comprehensive review was undertaken in order to arm the clinician-scientist with the most up-to-date evidence with which to evaluate the proposed and accepted mechanisms of modern SCS. More importantly, we sought to expose gaps in our understanding of these therapies and to identify potentially fruitful and unexplored avenues for future investigation. At this juncture, it is crucial to continue elucidating the mechanisms of P-SCS, PF-SCS, DRG-S and ECAP-SCS. Doing so may potentially lead to new or synergistic therapies for patients with debilitating chronic pain syndromes. Here, we reviewed the evidence and theory for the analgesic mechanisms of modern SCS. Specifically, we evaluated the modulation of 1) segmental and supraspinal neurotransmitters, 2) segmental and supraspinal neurophysiology/neuroplasticity, 3) central and peripheral neuroinflammation and 4) cortical and subcortical neurocircuits (Fig. 12). Moreover, further research is needed to characterize widely heterogenous pathophysiological processes that contribute to the progression and maintenance of chronic pain. These heterogenous pathological processes can evolve over time; however current SCS paradigms remain temporally fixed. Promising new SCS paradigms such as ECAP-SCS, DRG-S and the selective use of combined simultaneous P-SCS and PF-SCS may employ multiple, synergistic and adaptive mechanisms, thus opening the door to precision-based SCS aimed at specific pathogenic processes. In total, SCS is a safe and effective therapy for patients with neuropathic pain conditions and FBSS, which is currently undergoing rapid evolution. The clinical evidence supporting SCS is overwhelmingly positive while the level of evidence has steadily improved during the advent of HF-SCS, B-SCS and DRG SCS. With promising emerging paradigms such as ECAP-SCS and DRG-S as well as an arsenal of SCS therapies, the pain physician is responsible for making a weighty decision, the consequence of which may lead to an invasive procedure and subsequent device implantation for the patient. To substantiate this clinical choice, there is an urgent need to complete careful, systematic preclinical mechanistic and evidence-based clinical research to close our sizable knowledge gaps.

## Data Availability

Data sharing not applicable to this article as no datasets were generated or analyzed during the current study.

## Abbreviations

5-HT: 5-Hydroxytryptamine, Serotonin
A5: Noradrenergic Cell Group A5
A7: Noradrenergic Cell Group A7
ACh: Acetylcholine
AEN: Affective-emotional network
AI: Anterior Insula
AMPA: Amino-3-hydroxy-5-methyl-4-isoxazole propionate
Amy: Amygdala
AP: Action potential
ATF3: Activating transcription factor 3
BDNF: Brain-derived neurotrophic factor
CB: Charge-balancing
CC: Current-Controlled
CCI: Chronic constriction injury
c-Fos: Proto-oncogene
CGRP: Calcitonin Gene-related Peptide
CGRPR: Calcitonin Gene-related Peptide Receptor
CPM: Conditioned pain modulation
CRPS: Chronic Regional Pain Syndrome
CS: Central sensitization
CSF: Cerebrospinal Fluid
CXCL16: Chemokine C-X-C Motif Ligand 16
dACC: Dorsal Anterior Cingulate Cortex
DAS: Descending antinociceptive systems
DCML: Dorsal column medial lemniscus
DH: Dorsal Horn
DLF: Dorsal Lateral Funiculus
DM: Dura Mater
DMN: Default mode network
DNIC: Diffuse noxious inhibitory control
dPI: Dorsoposterior Insula
DRG-S: Dorsal Root Ganglion Stimulation
ECAP-SCS: Evoked Compound Action Potential-SCS
EEG: Electroencephalogram
EF: Electric Field
FBSS: Failed Back Surgery Syndrome
fMRI: Functional Magnetic Resonance Imaging
GABA: Gamma-aminobutyric Acid
GABAR: Gamma-aminobutyric Acid Receptor
GDNF: Glial Cell Line-Derived Neurotrophic Factor
GFAP: Glial fibrillary acidic protein
Glu: Glutamate
Gly: Glycine
GlyR: Glycine Receptor
HF-SCS: High Frequency Spinal Cord Stimulation
Hz: Hertz
IC: Insular cortex
IF: Interstitial Fluid
IFN-γ: Interferon Gamma
IL-10: Interleukin 10
Il-12: Interleukin 12
IL-13: Interleukin 13
IL-15: Interleukin 15
IL-17: Interleukin 17
IL-1β: Interleukin 1β
IL-2: Interleukin 2
IL-6: Interleukin 6
IL-8: Interleukin 8
INS: Insula
IPG: Implantable Pulse Generator
LC: Locus Coeruleus
LTP: Long Term Potentiation
MCP-1: monocyte chemoattractant protein 1
MEG: Magnetoencephalography
mGLU: Metabotropic glutamate
MOA: Mechanism of Action
NE: Norepinephrine
NGF: Nerve Growth Factor
NK1: Neurokinin-1
NK1: Neurokinin-1
NK1R: Neurokinin-1 Receptor
NMDA: N-methyl-D-aspartate
NpY: Neuropeptide Y
NS: Nociceptive Specific
OFC: Orbitofrontal cortex
PAG: Periaqueductal gray
PCC: Posterior Cingulate Cortex
PFC: Prefrontal Cortex
PF-SCS: Paresthesia Free Spinal Cord Stimulation
pgACC: Perigenual Anterior Cingulate Cortex
PHC: Parahippocampus
PN: Projection Neuron
PNS: Peripheral nervous system
P-SCS: Paresthesia Spinal Cord Stimulation
PW: Pulse width
QST: Quantitative sensory testing
RI: Rostral Insula
RIII: Flexor reflexes
RVM 5-HT Like: RVM 5-HT Like cells projecting from RVM to DH
RVM OFF: Rostral ventromedial medulla OFF cells
RVM ON: Rostral ventromedial medulla ON cells
RVM: Rostral ventromedial medulla
SC: Spinal Cord
SCS: Spinal Cord Stimulation
SDN: Sensory-discriminative network
SG: Substantia gelatinosa
SGC: Satellite glial cells
SI: Primary Somatosensory Cortex
SII: Secondary Somatosensory Cortex
SNI: Spared nerve injury
SNL: Spinal nerve ligation
SP: Substance P
SSEP: Somatosensory evoked potentials
STT: Spinothalamic tract
TCD: Thalamocortical dysrhythmia
TENS: Transcutaneous electrical nerve stimulation
TGF-β: Transforming Growth Factor Beta
TNF-α: Tumor Necrosis Factor Alpha
TrkB: Tropomyosin receptor kinase B
TS: Temporal summation
VC: Voltage-Controlled
VEGF: Vascular Endothelial Growth Factor
VIP: Vasoactive Intestinal Peptide
VLF: Ventrolateral Funiculus
VPL: Ventral Posterior Lateral nuclei of the thalamus
VPM: Ventral Posterior Medial nuclei of the thalamus
WDR: Wide Dynamic Range
α: Alpha
β: Beta
γ: Gamma
θ: Theta
